# Dicer-like proteins influence Arabidopsis root microbiota independent of RNA-directed DNA methylation

**DOI:** 10.1186/s40168-020-00966-y

**Published:** 2021-02-26

**Authors:** Richa Kaushal, Li Peng, Sunil K. Singh, Mengrui Zhang, Xinlian Zhang, Juan I. Vílchez, Zhen Wang, Danxia He, Yu Yang, Suhui Lv, Zhongtian Xu, Rafael J. L. Morcillo, Wei Wang, Weichang Huang, Paul W. Paré, Chun-Peng Song, Jian-Kang Zhu, Renyi Liu, Wenxuan Zhong, Ping Ma, Huiming Zhang

**Affiliations:** 1 Shanghai Center for Plant Stress Biology, CAS Center for Excellence in Molecular Plant Sciences, Chinese Academy of Sciences, Shanghai, 201602 China; 2 Department of Statistics, University of Georgia, Athens, GA 30602 USA; 3 University of Chinese Academy of Sciences, Beijing, 100049 China; 4 Current address: Haixia Institute of Science and Technology, Fujian Agriculture and Forestry University, Fuzhou, 350002 China; 5 Current address: Institute for Water Research and Department of Microbiology, University of Granada, Granada, Spain; 6 Shanghai Chenshan Botanical Garden, Shanghai, 201602 China; 7 Department of Chemistry & Biochemistry, Texas Tech University, Lubbock, TX 79409 USA; 8 State Key Laboratory of Crop Stress Adaptation and Improvement, Henan University, Kaifeng, 475004 China; 9 Department of Horticulture & Landscape Architecture, Purdue University, West Lafayette, IN 47906 USA

**Keywords:** Root microbiota, Microbiome, RNA-directed DNA methylation, DCL, Small RNA, Defense, Cell wall

## Abstract

**Background:**

Plants are naturally associated with root microbiota, which are microbial communities influential to host fitness. Thus, it is important to understand how plants control root microbiota. Epigenetic factors regulate the readouts of genetic information and consequently many essential biological processes. However, it has been elusive whether RNA-directed DNA methylation (RdDM) affects root microbiota assembly.

**Results:**

By applying 16S rRNA gene sequencing, we investigated root microbiota of Arabidopsis mutants defective in the canonical RdDM pathway, including *dcl234* that harbors triple mutation in the Dicer-like proteins DCL3, DCL2, and DCL4, which produce small RNAs for RdDM. Alpha diversity analysis showed reductions in microbe richness from the soil to roots, reflecting the selectivity of plants on root-associated bacteria. The *dcl234* triple mutation significantly decreases the levels of *Aeromonadaceae* and *Pseudomonadaceae*, while it increases the abundance of many other bacteria families in the root microbiota. However, mutants of the other examined key players in the canonical RdDM pathway showed similar microbiota as Col-0, indicating that the DCL proteins affect root microbiota in an RdDM-independent manner. Subsequently gene analysis by shotgun sequencing of root microbiome indicated a selective pressure on microbial resistance to plant defense in the *dcl234* mutant. Consistent with the altered plant-microbe interactions, *dcl234* displayed altered characters, including the mRNA and sRNA transcriptomes that jointly highlighted altered cell wall organization and up-regulated defense, the decreased cellulose and callose deposition in root xylem, and the restructured profile of root exudates that supported the alterations in gene expression and cell wall modifications.

**Conclusion:**

Our findings demonstrate an important role of the DCL proteins in influencing root microbiota through integrated regulation of plant defense, cell wall compositions, and root exudates. Our results also demonstrate that the canonical RdDM is dispensable for Arabidopsis root microbiota. These findings not only establish a connection between root microbiota and plant epigenetic factors but also highlight the complexity of plant regulation of root microbiota.

**Video abstract**

**Supplementary Information:**

The online version contains supplementary material available at 10.1186/s40168-020-00966-y.

## Background

Plants host a variety of soil microbes in the rhizosphere, where organic compounds are released from roots into the soil, resulting in a nutrient-rich environment for soil microbes [1–4]. The rhizosphere microbes are capable of influencing plants by different ways, such as through production of phytohormones that stimulate plant growth or pathogenic factors that cause disease symptoms in plants [5–9]. Although in vitro reconstruction of the rhizosphere microbiota for applications is still technically challenging, it has been increasingly clear that the rhizosphere bacteria community is important to plant vigor [10–12]. The assembly of rhizosphere microbiota is influenced by changes in the environmental factors such as soil humidity and iron nutrient availability [13–16], as well as by changes in plant immunity that governs plant responses to bacteria and other microbes [17–19]. In addition, the assembly of rhizosphere microbiota has also been shown to be affected by plant genotype [20–23]. However, it is unclear whether rhizosphere microbiota is influenced by plant epigenetic factors, which control genome stability and control the transcription of genetic sequences and thereby many important biological processes.

DNA methylation at the 5^th^ position of cytosine is a major epigenetic mark in plants. Through alterations in chromatin structure and the accessibility to transcription factors, DNA methylation alone or in combination with other epigenetic marks can influence transcriptional activities [24]. As a result, disruption of DNA methylation can lead to developmental abnormalities in plants as well as alterations in plant responses and adaptation to environmental changes [24–26]. In *Arabidopsis thaliana*, de novo DNA methylation is established through the RNA-directed DNA methylation (RdDM) pathway, in which complementary pairing between 24-nt small interfering RNAs (siRNAs) and nascent scaffold RNAs, together with protein–protein interactions, recruits the protein machinery for DNA methylation [24, 27]. In the canonical RdDM pathway, the production of siRNAs and scaffold RNAs depends on the plant-specific RNA polymerases Pol IV and Pol V, respectively. Pol IV generates single-stranded non-coding RNAs that are transcribed into double-stranded RNAs by RNA-dependent RNA polymerase 2 (RDR2) and are subsequently cleaved into 24 nt siRNAs by DCL-like protein 3 (DCL3). In addition, DCL2 and DCL4 that mainly generate 22 and 21 nt siRNAs, respectively, can function redundantly with DCL3 in mediating RdDM activities [28–30]. The complementary pairing between scaffold RNAs and siRNAs requires chromatin retention of Pol V-transcribed non-coding RNAs, and the process is facilitated by Rrp6-like 1 (RRP6L1), which is a putative 3′-5′ exoribonuclease that binds to and positively regulates some long non-coding RNAs [30]. RRP6L1 also positively regulated the accumulation of Pol IV-dependent siRNAs, likely through helping retain Pol IV-transcribed non-coding RNAs in the transcription complex for RDR2-dependent transcription [31, 32]. Dysfunction of some RdDM factors resulted in differential effects on plant susceptibility to pathogens [33–36], thus arousing the question whether RdDM is important to the assembly of rhizosphere microbiota.

In this study, we initially sought to characterize the role of canonical RdDM in shaping root microbiota, but unexpectedly found that root microbiota was not affected in the examined RdDM mutants except for *dcl234*, indicating that the function of DCL2/3/4 proteins in affecting RdDM-independent sRNAs is important for root microbiota. Specifically, we began with 16S rRNA gene sequencing to compare the root microbiota in RdDM mutants and wild type plants. After seeing that *dcl234* has different microbiota compared to the wild-type plants, we performed shotgun sequencing for their metagenomic DNA, in order to see whether there is any microbial gene whose abundance was significantly altered in the *dcl234*-associated microbes compared to the Col-0-associated microbes. The dcl234 metagenome showed altered abundance in some genes with defense- and metabolism-related functions, indicating that the *dcl234*-associated and the Col-0-associated microbial communities possibly were facing different plant defense and root metabolites. For this reason, we then performed mRNA sequencing to profile the plant transcriptome, which was highlighted by differential gene expression in several biological processes that could be important in plant-microbe interactions, such as defense, cell wall organization, and metabolism of pigments and other small molecules. Because the DCL2/3/4 proteins function in sRNA biogenesis, we also investigated sRNA transcriptome in order to find the sRNAs that may cause alterations in mRNA levels. Results of sRNA suggested that the altered mRNA levels of some genes, including several miRNA-targeted cell wall-related genes, could be correlated with altered sRNA homeostatsis. Subsequently, biological significance of differential gene expression was demonstrated by the decreased deposition of cellulose and callose in root xylem, as well as by the restructured profile of root exudates in *dcl234* compared with Col-0. Altogether, these results demonstrate an important role of the DCL proteins in shaping root microbiota through integrated regulation of plant defense, cell wall compositions, and root exudates.

## Materials and methods

### Plant materials and growth conditions

All *Arabidopsis thaliana* used in this study were in the Col-0 background. The single mutants *nrpd1*-*3*, *nrpe1*-*11*, *rrp6l1*-*1* and the triple mutants *ddc* (*drm1*-*2drm2*-*2cmt3*-*11*) and *dcl234* (*dcl2*-*1dcl3*-*1dcl4*-*2*) were previously described [28, 31, 37]. Seeds were sterilized by 100% ethanol for 1 min and then 20% house bleach solution for 15 min. After washing in sterile double-distilled water (dd H_2_O) for three times, the seeds were planted on 1/2-strength MS medium in round (0.7% agar) petri dishes. After stratification at 4 °C for 48 h, the seeds were placed in Percival CU36L5 growth chamber under long day conditions (16 h light/8 h dark cycle) at 21 ± 2 °C with 150 μmol photons m^−2^ s^−1^ light. Five-day-old seedlings were transferred to soil pots containing the mixed soil, at the rate of 5 seedlings per pot. The plants were then grown in a growth room with a 16:8 h day/night cycle, 22 °C during the day and 18 °C during the night at a relative humidity of 50–60%. The sampling for root microbiota analysis was done when seedlings were 21 days old. Unplanted pots were subjected to the same conditions as the planted pots to prepare the control soil samples at harvest.

The natural soil substrates were collected from the Chenshan Botanical Garden, Shanghai, China. The soil was cleaned from plant parts, worms and stones, and homogenized manually using a sieve (2.5 mm^2^). The cleaned and homogenized field soil was mixed with the commercial soil (Pindstrup Substrate) in 1:1 ratio. The mixed soil was then homogenized again and distributed to each pot. For each genotype and unplanted soil, samples from two pots (10 seedlings) were collected as one biological replicate. Four biological replicates were grown for each plant genotype. Details of the sample information are available in the Supplementary Table 1.

### Preparation of metagenomic DNA and 16s rRNA sequencing

The metagenomic DNA from soil, rhizosphere, and root samples were prepared as described by Bulgarelli et al. [38]. The plants along with the roots were removed from the soil and excess soil was manually shaken off the roots, leaving approximately 1 mm of soil still attached to the roots. The roots were harvested from 0.5 cm below the shoot-root junction. The roots were collected in 50 ml falcon tube containing 10 ml phosphate-buffered saline (PBS) solution (130 mM NaCl, 7 mM Na2HPO4, 3 mM NaH_2_PO_4_, pH 7.0, 0.02% Silwet L-77), and washed for 20 min at 180 rpm, then transferred to new tube and washed them in 3 ml PBS-S buffer for 20 min at 180 rpm again. The double-washed roots were transferred to new falcon tube and sonicated for 10 min at 160 W in 10 intervals of 30 s pulse and 30 s pause. After sonication, the roots were removed from PBS-S, rinsed in a fresh volume of 10 ml PBS-S buffer, and shortly dried on 50-mm-diameter Whatman filter paper (GE Healthcare USA), transferred to 2 ml tubes and frozen in liquid nitrogen for storage at − 80 °C. The soil suspensions collected in the falcon tubes were combined after the first and second washing treatments, centrifuged at 4000 g for 20 min, then the pellet was frozen in liquid nitrogen and store at − 80 °C until further processing. Two kind of soil samples were prepared viz. “Soil1”—the initial bulk soil (cleaned natural soil:Pindstrup substarte, 1:1), which was collected (100 g) at the time of transferring soil into the pots, frozen in liquid nitrogen, and stored at − 80 °C. “Soil2”—is the soil from unplanted pots which were subjected to the same conditions as the planted pots to prepare the control soil samples at harvest. The soil2 samples were collected (100 g) from the center of an unplanted pot after removing the top 0.5–1 cm of soil, frozen in liquid nitrogen, and stored at − 80 °C until further processing. The total DNA was extracted with the FastDNA® SPIN Kit for Soil (MP Biomedicals, Solon, USA) following the manufacturer’s instructions. The DNA samples were eluted in 100 μl DES water and the DNA concentrations were determined using Nanodrop 2000 (Thermo Fisher Scientific). Amplicon libraries were generated following the protocol of Illumina Miseq System for 16S metagenomic sequencing library preparation. The PCR primer sequences 799F (5′-AACMGGATTAGATACCCKG-3′) [39] and 1193R (5′-ACGTCATCCCCACCTTCC-3′) [40], which span ~ 400 bp of the hypervariable regions V5–V7 of the prokaryotic 16S rRNA gene, were used during the first-round PCR to amplify the V5–V7 regions of 16S rRNA genes. PCRs were performed on 96-well plate with KAPA HiFi HotStart ReadyMix using 2.5 μl of 5 ng/μl adjusted template DNA in a total volume of 25 μl. PCR components in final concentrations included 12.5 μl of 2× KAPA HiFi HotStart ReadyMix, 5 μl of 1 μM of each fusion primer. The PCR reactions were assembled in a laminar flow and amplified using protocol; 95 °C for 3 min, 25 cycles of 95 °C for 30 s, 55 °C for 30 s, 72 °C for 30 s, and extension at 72 °C for 5 min. For each biological replicate, there are three technical replicates. Triplicate reactions of each sample were pooled and a 5 μl aliquot inspected on a 2% agarose gel. The PCR primers 799F and 1193R produce a mitochondrial product at ~ 800 bp and a bacterial amplicon at ~ 400 bp. The bacterial amplicon was extracted from the gel with sharp scalpel and eluted from the agarose using the QIAquick Gel Extraction kit (QIAGEN, Hilden, Germany). Following purification and elution in sterilized double-distilled water, the concentration of the amplicon DNA in each sample was determined by using Qubit™ dsDNA HS Assay Kit on Qubit®2.0.

The first-round PCR products were further barcoded during the second-round PCR following the protocol of Illumina Miseq System for 16S metagenomic sequencing library preparation. The 2^nd^ PCR amplification used unique barcode and indexed sequencing adaptor sequences (Supplementary Table 1). About the index primer, the forward primer was 2P-F (Supplementary Table 1) and the sequencing index is embedded in the reverse primer, 2P-R (Supplementary Table 1). The second amplification was conducted in 20 μl volume containing 1 μl nuclease-free water, 1.6 μl dNTP, 2 μl of buffer, 10 μl of Taq Master Mix, 0.2 μl of primer FP (10 μM), 0.2 μl of primer RP (10 μM), 2 μl of primer 2P-F (10 μM), 2 μl of primer 2P-R (10 μM), and 1 μl of first round PCR product. The PCR was run with the conditions: 95 °C for 3 min, 10 cycles of 95 °C for 30 s, 55 °C for 30 s, 72 °C for 1 kb/min, and extension at 72 °C for 5 min. Four biological replicates of each genotype were successfully prepared except for Col-0 and *nrpd1*-3 that had three instead of four biological replicates. Samples were sequenced at the Genomics Core Facility at Shanghai Center for Plant Stress Biology, Shanghai, China.

### 16S rRNA gene sequencing data analysis

For data analysis, the raw data and quality was controlled and preprocessed using FastQC v0.11.8 and trimmomaticv0.36 [41], and the processed high quality data was assembled with FLASH v1.2.11 [42], requiring an overlap of at least 10 bp (-m 10) for the two paired-end reads. The assembled sequences were demultiplexed by using the extract_barcodes.py script to extract the barcode sequence file. We then used split_libraries_fastq.py with the mapping file, the barcode file and our data as the input to demultiplex the samples. For subsequent analysis, we mainly used QIIME v1.91software [43]. The chimeric sequences were removed using the usearch (-m usearch61) method of the script identify_chimera_seqs.py with “Gold” database (-r gold.fa). The remaining high-quality sequences were clustered using the script pick_open_reference_otus.py and OTUs (operational taxonomic units) were classified. We used assign_taxonomy.py script with the UCLUST algorithm as default and Greengenes13_8 at 97% identity as the reference database to do taxonomic classification. A sequence with more than 97% identity is clustered, and only at least 2 sequences in a cluster were output. The OTUs belonging to mitochondria, *Chlorophyta*, *Archea*, and *Cyanobacteria* were then removed using the custom script. By this way, a total of 6,593,329 high-quality sequences were obtained with a median read count of 130007.5 per sample (range 23,421–208,621). The high-quality reads were clustered into 10,850 microbial OTUs.

Next, we used the function calculateRarefaction of the R package ShotgunFunctionalizeR [44] to evaluate the rarefaction curve. Then we used the script multiply_rarefaction.py of QIIME to generate the rarefied tables (100× tables from 23,000 sequences per sample, step of 230 sequences) and generated a table composed of 8602 OTUs as the threshold-independent community (TIC) [22]. Among the TIC, a minimum of 20 sequences per OTU in at least one sample was used as a criterion to define abundant community members (ACM) [22]. The relative abundance (RA) of each ACM in a sample was calculated by dividing the reads of the ACM by the sum of the usable reads in that sample. The relative abundance of each phylum or family was the sum of the ACMs belonging to that phylum or family in a sample. The significant differences between samples were assessed by the ANOVA-based statistics with Tukey’s HSD test. The UniFrac distance was calculated using the script beta_diversity.py to evaluate beta-diversity between samples based on the ACM. Heatmaps were performed by the online tool of iDEP.90 (http://bioinformatics.sdstate.edu/idep/).

### Shotgun sequencing of the metagenomic DNA

The metagenome sequencing of Col-0 and *dcl234* root samples used the same DNA samples as the 16S rRNA gene sequencing. The genomic DNA (500 ng) was first sheared by a Covaris M220 Focused-ultrasonicator to 200 bp fragments. Fragmented DNA was then used for performing the library preparation with NEBNext Ultra II DNA Library Prep Kit from Illumina (New England BioLabs, E7645L) according to the manufacturer’s instructions with different barcodes. The prepared libraries were assessed for quality by using NGS High-Sensitivity kit on the Fragment Analyzer (AATI) and for quantity by using Qubit 2.0 fluorometer (Thermo Fisher Scientific). All libraries were sequenced in paired-end 150 bases protocol (PE150) on an Illumina HiSeq sequencer.

Raw paired-end Illumina reads were processed using Trim Galore [45] through custom scripts for adapter removal, ambiguity, length, and quality trimming with the default setting. Each sample’s high-quality reads were mapped to the Arabidopsis reference genome using STAR [46] with customized for DNA alignment settings. The unmapped reads were kept and assembled into contigs using the MEGAHIT assembler [47] with default parameters. The contigs longer than 1000 bp were binned by a reference-free metagenomic binning pipeline MetaGen [48]. Eighty-eight bins were found, and the contigs in every bin were mapped to the NCBI RefSeq database. Prokka [49], which is a command line software tool for annotating all relevant genomic features on a draft bacterial genome, was used to identify and annotate the protein-coding genes in the assembled DNA sequences. To quantify the genes, we mapped the input reads back to the assembly. The coverage of each gene predicted and annotated by Prokka was calculated using Bedtools v2.28.0 [50] and the scripts prokkagff2bed.sh and get_coverage_for_genes.py after the raw reads were mapped back to the assembled contigs. These scripts were developed by the Environmental Genomics group at SciLifeLab Stockholm. We then used the gene-wise exact tests [51] to examine the difference in mean abundance between the two groups of interest and got a *p* value for each region. The tests were performed using EdgeR [52] package in R language. We used the Bonferroni and FDR method to do multiple testing correction and achieved the adjusted *p* values.

### Whole genome sequencing of small RNAs and data analysis

Total RNA was extracted from shoots of soil-grown 21-day-old plants of Col-0 and *dcl234* by using the Trizol reagent (Ambion, USA). Library construction and deep sequencing were performed at the Genomics Core Facility of Shanghai Center for Plant Stress Biology, CAS, China. Briefly, small RNA was extracted after gel electrophoresis of the total RNA. For small RNA sequencing, NEBNext Multiplex Small RNA Library Prep Kit for Illumina was used and libraries were sequenced by paired-end 150 bases protocol (PE150) on an Illumina HiSeq 2500 sequencer. Analysis of small RNA data was conducted according to Zhang et al. [31]. The adapters were removed from raw sRNA sequences by Cutadapt v1.12 (--discard-untrimmed) [53]. The raw reads without the adapter sequence were removed, as sRNA sequences are short and so if a sequence was obtained without the associated adapter sequence, the read should not be trusted. Clean reads with a sequence length of 18–60 nt were extracted using seqkit v0.7.2 [54]. After structure RNAs were removed, the remaining reads were then aligned to the Arabidopsis genome (TAIR10) by Bowtie v1.2.2 [55]. The table of sRNA counts was obtained by custom script. sRNA with reads greater than 5 in any sample was retained.

The “hits-reads count” (HRC) values were calculated by dividing the reads count for each small hit, where a hit is defined as “The number of loci at which a given sequence perfectly matches the genome” [56]. sRNAs HRC>10 in any sample were used to identify the differentially expressed sRNA by edgeR [52]. The “hits-normalized-abundance” (HNA) values were calculated by dividing the normalized abundance (in RPTM) for each small RNA hit. The HNA values of all sRNAs with individual non-overlapping 500-nt windows throughout the whole genome were compared between the mutant and wild type. Each window of HNA values was summed and a cutoff of 400 was applied. Windows between the wild-type and mutant samples show ≥ 2 and ≤ 0.5-fold HNA value change were identified as the “differentially expressed sRNA region” (DESR). We used bedtools v2.25.0 [50] to annotate the distribution of DESR on the genome. The miRBase [57] was used to manually annotate and confirm the identified miRNA in sRNA dataset by using the default values. The miRNA targets were identified in the Plant MicroRNA Database [58].

### mRNAseq and data analysis

Total RNA were extracted from shoots of the same plants whose roots were used for the microbiota experiments, in order to show transcriptomes of the plants that were exactly associated with the observed microbiota. The mRNA was isolated from the total RNA by using NEBNext Poly(A) mRNA Magnetic Isolation Module. The NEBNext Ultra II Directional RNA Library Prep Kit was used for library construction. The library was sequenced in paired-end 150 bases protocol (PE150) on an Illumina HiSeq 2500 sequencer. We used trimmomatic1 v0.36 [41] to perform data preprocessing on the paired-end reads. Clear reads obtained after trimming the adapter sequence, removing low-quality bases, and filtering short reads were used for subsequent analysis. Clean reads were mapped to the *Arabidopsis thaliana* genome (TAIR10) by using HISAT v2.1.0 [59] with default parameters. The number of reads mapped to each gene was calculated using the htseq-count script in the software HTSEQ v0.9.1 [60]. The differentially expressed genes were identified by edgeR [52]. Genes that showed at least 1.5-fold change in expression and FDR ≤ 0.05 are considered to be differentially expressed genes (DEGs).

### Quantitative real-time PCR

The plants were grown in half MS medium with 0.7% agar. Five- and ten-day-old seedlings were collected in 2 ml tubes and frozen in liquid nitrogen. After homogenization, total RNA was extracted using the Trizol reagent and quantified by using the NanoDrop 2000 (Thermo Fisher Scientific). For mRNA expression analysis, 1 μg total RNA was used for reverse transcription with oligo dT using the Superscript III RT kit (Invitrogen) according to the manufacturer’s instructions. The qRT-PCR was performed with iTaq Universal SYBR Green PCR master mix (Bio-Rad) on a CFX96 real-time PCR detection system (Bio-Rad). Each 20 μl reaction mixture was composed of 10 μl SYBR Green (BioRad), 1 μl of 10 μM primers (forward and Reverse), 6 μl deionized H_2_O, and 2 μl cDNA. The qPCR cycling condition was set at initial denaturation of 30 s at 95 °C followed by 40 cycles of denaturation at 95 °C for 10 s and annealing at 60 °C for 45 s. After the completion of amplification, melt curve was produced to determine the primer specificity at 65 °C for 5 s, followed by heating up to 95 °C with 0.5 °C increment. Relative transcript levels were calculated using the comparative delta-Ct method and normalized to the transcript levels of ACTIN2 (At3g18780). The primers used in this study are listed in Supplementary Table 2.

### Plant cell wall staining and microscopy

To stain cellulose, Mitra and Loque [61] protocol for Calcofluor staining was followed with some modifications. Briefly, the roots were transferred to 2 ml tubes and stained with 0.02% calcoflour white (Sigma) for 5 min. The primary root was placed on the microscope glass slide and transverse section of middle part of the roots was prepared using sharp blade. Calcofluor White was visualized using an epifluorescence microscope (Zeiss Imager M2).

The staining of callose was done according to Muller et al. [62] with some modifications. Briefly, the roots were stained with 0.1% (w/v) aniline blue solution in 0.1 M sodium phosphate buffer (pH = 7.2) for 1.5 h. The cross-section of roots was done as described above and visualized under epifluorescence microscope (Zeiss Imager M2).

### Measurement of cellulose contents

To determine the cellulose contents, the method involving sulfuric acid and nitric acid was used as described in Brux et al. [63]. Briefly, seedlings were kept at 65 °C in 90% ethanol for 30 min to inactivate the enzymes, followed by drying the alcohol-insoluble materials overnight at 80 °C. The hemicellulose and pectin materials were removed by boiling the dried material in a mixture of 73% acetic acid and 9% nitric acid. The boiled material were centrifuged and washed with water followed by acetone. The cellulose was dissolved in 72% sulfuric acid, and the resulting glucose concentration was measured spectrophotometrically at 650 nm after adding 3% anthrone in sulfuric acid.

### Quantification of anthocyanin levels

Anthocyanin levels were determined following the protocol described in Morcillo et al. [18]. Briefly, roots from three Arabidopsis plants of 18 days after germination were pooled and the fresh weight was measured followed by fine grinding with liquid nitrogen. The extraction buffer (45% methanol, 5% acetic acid) was proportionally added to per unit of fresh weight of the tissues and mixed thoroughly. Two rounds of centrifugation was done to remove the debris, at 12,000×*g* for 5 min at room temperature. The absorbance of the supernatant was measured at 530 and 637 nm, using a Microplate Reader Thermo Varioskan Flash. 13. Anthocyanin contents (Abs530/g F.W.) were calculated by [Abs530 − (0.25 × Abs657)] × volume added.

### Root exudate collection

Samples were prepared following a set up based on Ziegler et al. [64] with some modifications. The bottom of 96-well PCR plate were clipped off (4 mm) before sterilization, and were kept in an apparatus filled with 0.75% Agar to block the open end for filling the well with 0.5 MS medium with 1% sucrose. After solidification of medium, one Arabidopsis seed per well were sown with the help of a pippete tips. For mock, the similar plate without seeds was prepared. The 96-well PCR plates were fitted in 96-well deep well block containing 2.0 ml of liquid half MS medium without sucrose in each well. The cut end of each 96-well PCR plate was immersed about 2–3 mm into the medium. The wells at all the four corners were kept empty to fit with PCR tubes (200 μl) without cap and a sterile lid of 96-well plate were used to cover from above. The blocks were incubated in dark for 48 h at 4 °C for stratification followed by moving them to growth chamber at under long day conditions (16 h light/8 h dark cycle) at 21 ± 2 °C with 200 μmol photons m^−2^ s^−1^ light. After 5 days when the primary roots penetrated the MS plugs, the PCR plates containing the seedlings were transferred to a new 96-well deep well block with fresh sucrose free 2.0 ml of 0.5 MS medium, taking care to immerse each root in the corresponding well. After another 10 day of growth under the same growth conditions, the medium was collected, filtered through 0.25 μm Milipore filter, flash frozen, and freeze dried by lyophilization. The samples were kept at − 80 °C until further processed. At least three biological replicates were used for each genotype and 0.5 MS without sucrose was used as a control. One 96-well plate was considered as one biological replicate.

### Gas chromatography-Mass spectrometry analysis

Metabolite derivatization was performed as described [65] with minor amendments based on Barsch et al. [66]. Briefly, the lyophilized samples were dissolved in 1 ml of 80% methanol and the solution was then derivatized by addition of 100 μl methoxylamine hydrochloride in pyridine (20 mg/ml) for 70 min at 37 °C and with 100 μl *N*-methyl-*N*-(trimethylsilyl)trifluoroacetamide (MSTFA) for 30 min at 70 °C. Samples were continuously mixed during process.

The derivatized root exudates were analyzed by using a 7890B GC System (Agilent 7000 Series Triple Quad GC/MS) gas chromatographer (Agilent Technologies, Inc., CA, USA) based on Gorzolka et al. [67] with some modifications. Samples of 1 μl were injected and evaporated at 250 °C in the splitless mode and separated on a 30 m × 0.25 mm DB-5MS column with DuraGuard (+ DG) with 0.25 μm coating of phenyl arylene polymer (Agilent Technologies, Inc., CA, USA). The Helium carrier-gas flow was adjusted to 1 ml/min. The interphase temperature was set to 250 °C and the ion source temperature to 200 °C. Oven temperature was kept constant for 3 min at 80 °C and subsequently raised to 300 °C at 3 °C/min. To equilibrate the system, an incubation of 2 min at 80 °C was applied after each analysis. Mass spectra were recorded at 2 scan/s with a scanning range of 50 to 550 m/z. Metabolites were identified by comparison with purified standards, the NIST 2017 database (NIST, Gaithersburg, MD, USA), and the Golm Metabolome Database (GMD) [68, 69] for compound mass spectra patterns and chromatographic retention time. Peak areas of the identified metabolites were automatically quantified by using the processing setup implemented in Xcalibur™ v1.4 software (Thermo Fisher Scientific, MA, USA) and the MassHunter Software Package (Mass Profiler Professional (MPP) software; Agilent Technologies, Inc., CA, USA). The peak areas were then normalized by the peaks of pyridine and the dry weight of the sample. In addition to the samples, three blank samples of pyridine were also run to compare with the pyridine peaks in each sample. Experiments were performed with three independent biological replicates.

## Results

### Characterization of the underground bacteria communities in different compartments

To investigate whether dysfunction in the RdDM pathway affects root-associated microbial communities, we first performed 16S rRNA gene sequencing to identify the microbiota in three compartments, namely bulk soil, rhizosphere, and roots, by following the protocol reported previously [20, 22]. The wild-type Arabidopsis accession Col-0 was compared with five RdDM mutants including *nrpd1*-*3* that is defective in Pol IV, *nrpe1*-*11* that is defective in Pol V, *ddc* that is defective in the DNA methyltransferases DRM1, DRM2, and CMT3, *rrp6l1*-*1* that is defective in RRP6L1, and the triple mutant *dcl234*. The plants were grown for 21 days under controlled environmental conditions in soil substrates collected from Chenshan Botanical Garden located in Shanghai, China.

The hypervariable regions V5-V6-V7 in the prokaryotic 16S rRNA gene were selectively amplified [22], by using the DNA preparations from 8 bulk soil, 22 rhizosphere, and 22 root samples. A total of 10999 unique prokaryotic operational taxonomic units (OTUs) were identified from all the samples (see “Materials and Methods” for experimental procedures). The abundant community members (ACMs) were defined with a minimum threshold of 20 sequences per OTU in at least one sample [22, 38]. The ACMs, including bulk soil, rhizosphere, and root samples, were represented by 678 bacterial OTUs comprising 82.14% of rarefied quality sequences (Supplementary Table 3).

**Fig. 1:**
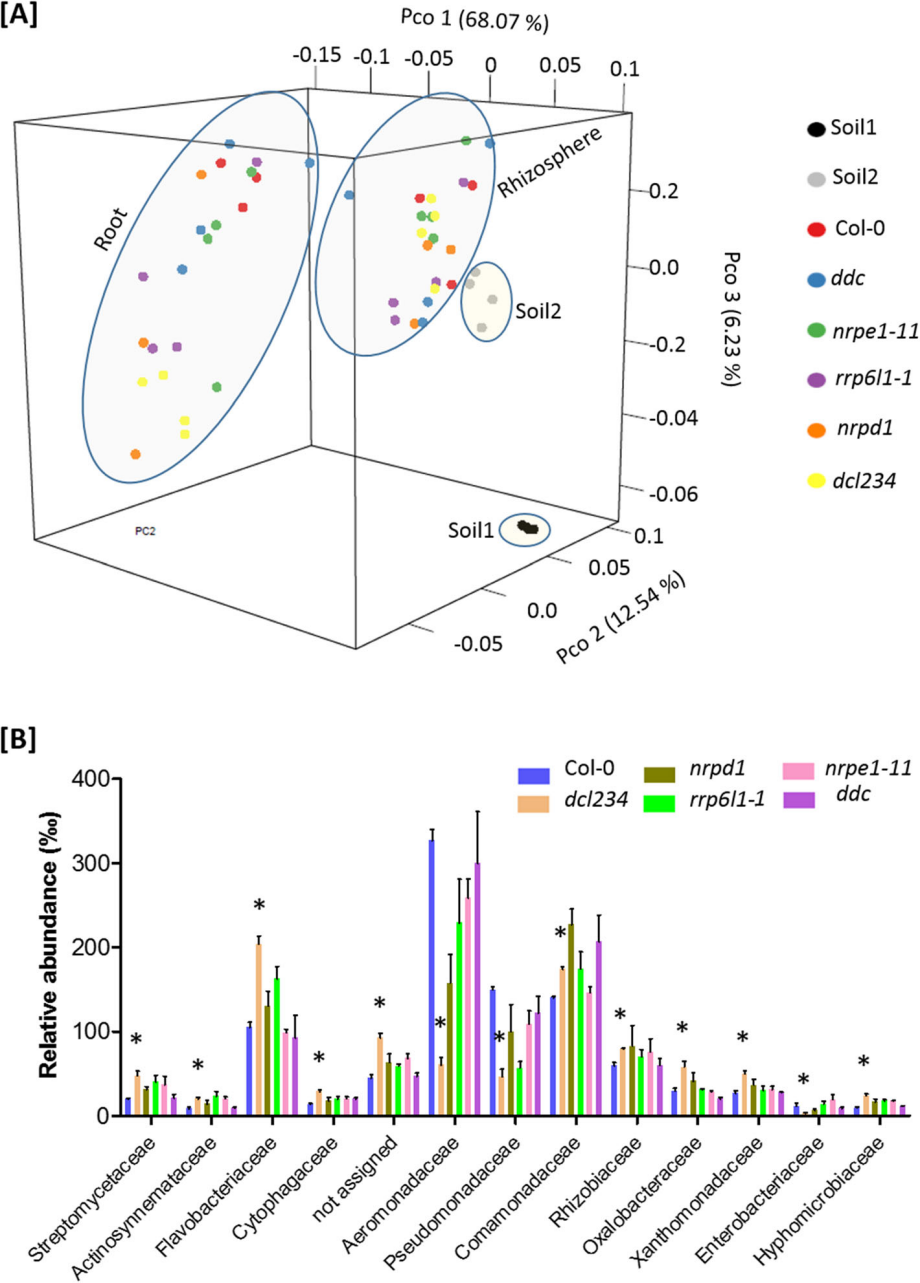
Arabidopsis root-associated microbiota is altered in the *dcl234* mutant. **a** PCoA using weighted UniFrac metrics indicates that the largest separation between bacterial communities is spatial proximity to the root (PCo 1). The bacteria communities were investigated by 16S rRNA gene sequencing of the metagenomic DNA. *n* ≥ 3 biological replicates. **b** Relative abundance of the top 13 abundant families in roots of the wild type Arabidopsis (Col-0) and the RNA-directed DNA methylation pathway mutants. Mean ± SE, *n* ≥ 3. Asterisks indicate significant difference (FDR ≤ 0.05) between the mutant and the wild type. Taxa with RA > 5‰ in at least one sample were included in the statistical analysis. The bacteria communities were investigated by 16S rRNA gene sequencing of the metagenomic DNA. See also Figures S1, S2, S3, S4

To determine the most influential factor to the bacterial community assembly, we analyzed weighted UniFrac distances between samples by performing principal coordinate analysis (PCoA). The bacteria communities showed clustering patterns based on the sample compartments (Fig. 1a). The initial bulk soil samples (soil 1), which were collected before planting, differed from the final bulk soil samples (soil 2) that were collected at sample harvesting, because the former clearly clustered away from the rhizosphere samples, whereas the latter showed only a minor separation from the rhizosphere samples. Importantly, the clustering of root samples was clearly separated from soil 2, and to a less degree, from the rhizosphere samples, along the 1^st^ and the 2^nd^ principal coordinates, which accounted for 68.07% and 12.54% variations, respectively (Fig. 1a, Figure S1). Therefore, the weighted UniFrac analysis identified the compartment as the major factor that influences the assembly of microbiota.

To gain more insights into the effects of compartments on the microbiota assembly, we compared the richness of OTUs in bulk soil and the plant-associated microhabitats. Alpha diversity analysis showed that the total numbers of OTUs, including either the observed OTUs or the estimated OTUs obtained by the Chao1 estimator, were much greater in the bulk soil samples than the root samples, while the rhizosphere samples showed similar but lower numbers of OTUs compared to the soil samples (Figure S2A, B). Similar patterns were observed when the Shannon index was applied to the analysis for community richness (Figure S2C). Together, the reductions in bacteria richness from the soil to roots reflect the selectivity of plants on root-associated bacteria.

We next analyzed the taxonomic structure of ACMs at the phylum level with the index of relative abundance (RA), which was calculated by dividing the reads of an OTU in a sample by the total reads in the same sample. Among all the detected phyla, *Proteobacteria* was the most abundant phylum irrespective of either compartment or genotype (Figure S3A-C; Supplementary Table 3). Firmicutes was the second most abundant (159‰) phylum in the initial bulk soil, but was almost the lowest abundant in the final bulk soil, rhizosphere, and root samples. In contrast, several other phyla showed increased abundance, for instance, *Bacteroidetes* and *Gemmatimonadetes* increased from 130‰ and 23‰ in the initial bulk soil to 210‰ and 35‰ in the final bulk soil, respectively. Compared to the final bulk soil, rhizosphere showed a reduction in *Actinobacteria*, meanwhile maintaining similar patterns of the overall phyla composition, which was mainly represented by *Proteobacteria*, *Bacteroidetes*, *Actinobacteria*, *Acidobacteria*, *Verrucomicrobia*, and *Gemmatimonadetes* (Figure S3B). In roots, the bacteria community was mainly represented by the phyla *Proteobacteria*, *Bacteroidetes*, and *Actinobacteria*, whereas the relative abundance of *Acidobacteria*, *Verrucomicrobia*, and *Gemmatimonadetes* was significantly reduced compared to that in the rhizosphere (Figure S3C). Therefore, these results further demonstrate the selectivity of plants on root-associated microbes.

### Root microbiota is altered in *dcl234* but not the other examined RdDM mutants

After characterizing the common effects of plants on soil bacteria assembly, we compared each of the RdDM mutants with the wild-type plants (Col-0) in a pairwise manner for their associated microbiota. Each mutant showed a similar rhizosphere bacteria community as Col-0 (Figure S3B); meanwhile, root microbiota was altered in *dcl234* but not the other examined mutants compared to Col-0 (Fig. 1b; Figure S3C), thus demonstrating a unique role of the DCL proteins in shaping Arabidopsis root microbiota in a way that is independent of their functions in mediating RdDM.

Three of the top five enriched bacteria phyla in root microbiota showed statistically significant (FDR ≤ 0.05) differences between *dcl234* and Col-0, including *Actinobacteria* and *Bacteroidetes* that were more enriched as well as *Proteobacteria* that was the most enriched phylum in Col-0 but became less enriched in *dcl234* (Figure S4A). A total of 23 differentially enriched (FDR ≤ 0.05) OTUs (deOTUs) were identified in *dcl234* compared to Col-0 (Figure S4B). At the phylum level, these deOTUs are composed of *Actinobacteria* (2), *Bacteroidetes* (3), and *Proteobacteria* (18). Most of the deOTUs showed increased enrichment in *dcl234*; meanwhile, decreased enrichment was observed exclusively in 6 *Gammaproteobacteria* deOTUs, which can be further identified to the family level (*Aeromonadaceae*) or the genus level (*Pseudomonas*). At the family level, *dcl234* showed increased (FDR ≤ 0.05) enrichment in the *Streptomycetaceae*, *Flavobacteriaceae*, *Comamonadaceae*, *Oxalobacteriaceae*, *Rhizobiaceae*, and *Xanthomonadaceae* families, each of which accounted for at least 5% in the *dcl234* root microbiota assembly (Fig. 1b). On the other hand, the relative abundance of *Aeromonadaceae* and *Pseudomonadaceae*, the top two most enriched families in Col-0, were drastically decreased in *dcl234* with statistical significance (FDR ≤ 0.05) from 327 to 61‰ and from 150 to 46‰, respectively (Fig. 1b). These results collectively demonstrate an influential role of the DCL proteins in the assembly of Arabidopsis root microbiota.

### Root-associated microbial metagenome implies altered plant-microbe interactions by the *dcl234* mutation

To gain further insights into the impacts of the *dcl234* mutations on root-associated microbiota, we sought for microbial gene-related information by performing metagenome sequencing of the *dcl234* and Col-0 root samples. The same DNA preparations for 16S rRNA gene amplification were used and generated more than 23 million total reads. After removal of reads from plant DNA, comparison between *dcl234* and Col-0-associated microbial DNA identified 7 differentially abundant genes (*p* < 0.05, FDR; Table 1), including 4 and 3 genes that are more and less enriched, respectively, in the *dcl234* associated microbiome compared to that of Col-0. Notably, two aminoglycoside 3′-phosphotransferases genes, *neo* and *aphA*, were identified as more enriched in *dcl234* roots than Col-0 roots. Aminoglycoside 3′-phosphotransferases are known to confer bacteria resistance to various aminoglycoside antibiotics [70]. Thus, the results implied that the microbes dwelling in *dcl234* roots might be facing a more defensive environment than that in Col-0 roots. Interestingly, microbial DNA accounted for 32.6 % of all sequencing reads in Col-0 roots, whereas the same proportion was 18.0% in *dcl234* roots (Figure S5). Given that the total reads in Col-0 and *dcl234* roots are similar (Figure S5), the significant reduction (*p* < 0.05, *t* test) in the proportion of microbial DNA seems to also indicate that the microbes associated with *dcl234* roots might be facing a more challenging environment compared to those with Col-0 roots.

**Table 1:** Microbial genes that are differentially abundant in *dcl234* and Col-0 root microbiomes

Protein	Name	Function (Uniprot)	logFC (dcl234/Col-0)	*p* value	EC_ number
Beta-glucuronidase	*uidA*	Carbohydrate metabolic process	7.91	1.05E-31	3.2.1.31
Alanine dehydrogenase	*AlaDH*	Oxidation-reduction process	10.75	8.4E-105	1.4.1.1
Aminoglycoside 3′-Phosphotransferase	*neo*	Kanamycin-kinase, antibiotic activity	11.21	1.3E-150	2.7.1.95
Aminoglycoside 3′-Phosphotransferase	*aphA*	Kanamycin-kinase, antibiotic activity	9.52	3.35E-52	2.7.1.95
Ornithine carbamoyltransferase	*argF*	Arginine biosynthetic process via ornithine; citrulline biosynthetic process	− 3.30	1.09E-06	2.1.3.3
2-Keto-4-carboxy-3-hexenedioate hydratase	*ligJ*	Lignin catabolic process	− 3.15	2.52E-07	4.2.1.-
Glutamyl-tRNA(Gln) amidotransferase subunit A	*gatA*	Aspartyl-tRNA aminoacylation	− 3.33	5.23E-07	6.3.5.7

Compared to Col-0 root microbiome, the *dcl234* root microbiome also showed altered abundance of several metabolism-related microbial genes, including the more abundant *AlaDH* that encodes an alanine dehydrogenase whose function is central to metabolism in microorganisms [71], the more abundant *uidA* that encodes a β-glucuronidase functioning in carbohydrate metabolic processes [72], and the less abundant *ligJ* that encodes a 2-keto-4-carboxy-3-hexenedioate hydratase functioning in lignin catabolic processes [73]. Together, these metagenomic results further elucidate the influential role of the DCL proteins in root microbiota, as well as indicate that the alterations in microbiota may result from alterations in plant defense- and metabolism-related processes.

### Genome-wide profiling of mRNAs and small RNAs highlighted *dcl234*-altered biological processes that are important for plant-microbe interactions

Since the DCL proteins function in small RNA (sRNA) production, we deduced that the disrupted sRNA homeostasis in *dcl234* alters the expression levels of certain genes and thereby alters plant interaction with root microbiota. Thus, we next investigated the plant mRNA transcriptome and its potential linkage with the sRNA homeostasis that is dependent on these three DCL proteins. RNAseq profiling of *dcl234* in comparison with Col-0 identified a total of 3090 differentially expressed genes (DEGs; fold change ≥ 1.5, *p* ≤ 0.05; FDR), including 1482 upregulated and 1608 downregulated genes (Table S4).

**Fig. 2:**
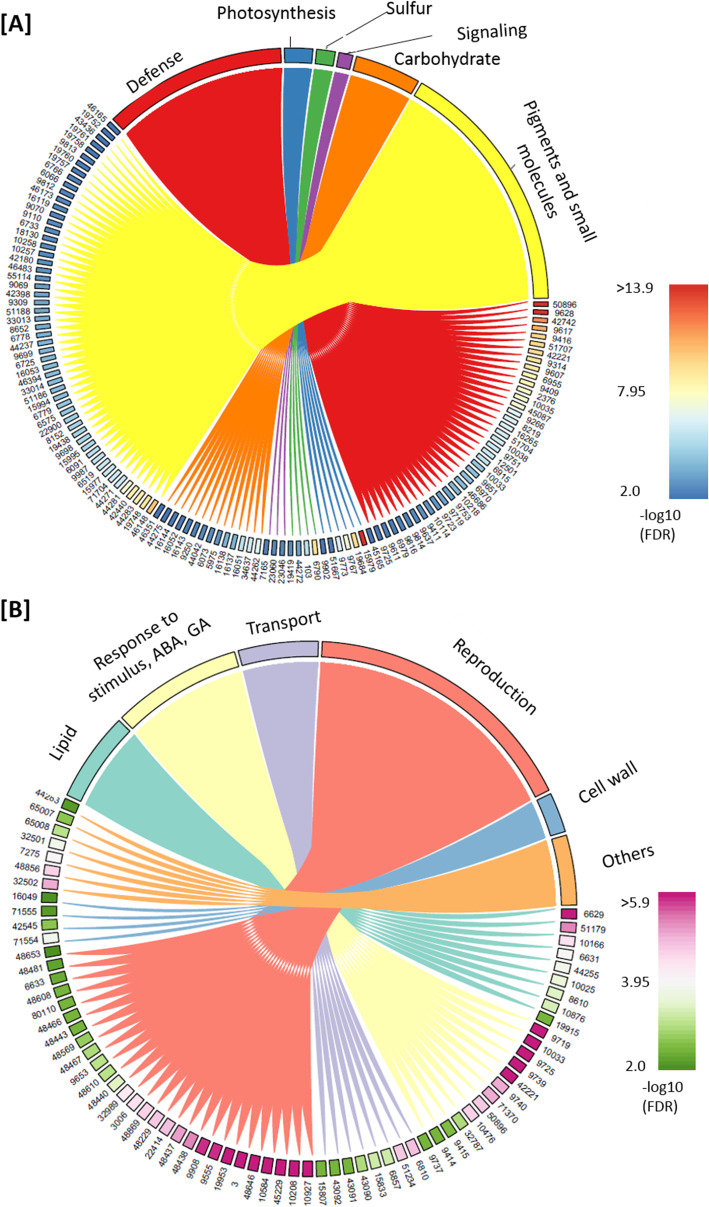
Genome-wide profiling of mRNAs highlight *dcl234*-altered biological processes that are important for plant-microbe interactions. The differentially expressed genes (DEGs; fold change ≥ 1.5, FDR ≤ 0.05, *n* = 4) that were upregulated (**a**) and downregulated (**b**) in *dcl234* compared to Col-0 were subject to the gene ontology (GO) analysis. The chord diagrams show the GO terms that link to their sub-classifications. The sub-classifications are labeled with GO ID that can be queried together with their corresponding DEGs in Table S4. GO analysis were performed by Bingo analysis of Cytoscape software at *p* ≤ 0.01 level of significance representing the *p* value cut-off of over-representation equal or less than the cutoff for each GO category. See also Figure S6

Gene ontology (GO) analysis of the upregulated DEGs highlighted two major clustering, which are composed of either defense-related genes or genes involved in the metabolic processes of pigments and other small molecules (Fig. 2a; Table S4). Within the clustering of defense-related DEGs, subgroups of genes involved in plant responses to bacteria, fungi, salicylic acid (SA), or jasmonic acid (JA) coexist with other immune response genes (Table S4), indicating that the *dcl234* mutations resulted in activation of plant defense responses. In Arabidopsis root microbiota, exogenous SA enhances *Flavobacerium* accumulation while SA mutants showed depletion of *Stretomyces* and *Pseudomonas* spp. [17]. In addition, activation of JA signaling also depletes *Pseudomonas* sp. in Arabidopsis root microbiota [74]. Thus, the activation of defense responses in *dcl234* may account for both the increased enrichment of *Flavobacteriaceae* and the decreased enrichment of *Pseudomonadaceae*. Within the clustering of pigments and small molecules, the subgroups include the biosynthetic processes of secondary metabolites, such as phenylpropanoid, flavonoid, and glucosinolate (Table S4), which are known to mediate plant defense responses or plant interactions with microbes [16, 75, 76]. Consistent with the upregulation in flavonoid biosynthesis, *dcl234* plants showed elevated anthocyanin accumulation in the roots (Figure S6B). In addition, GO analysis of the *dcl234* upregulated DEGs also highlighted a group of genes involved in sulfate reduction and sulfate assimilation (Fig. 2a; Figure S6C); this appears consistent with the upregulated biosynthesis of glucosinolates, which are sulfur-rich metabolites found mainly in the *Brassicaceae* and can play important roles in plant defense [75, 77]. Together, these results revealed that the *dcl234* mutations cause alterations in plant defense-related processes that would lead to altered plant interactions with root microbiota.

Meanwhile, GO analysis of the *dcl234* downregulated DEGs highlighted several biological processes, including lipid biosynthesis, responses to ABA or GA, peptide transport, reproduction, and cell wall organization (Fig. 2b).

**Fig. 3:**
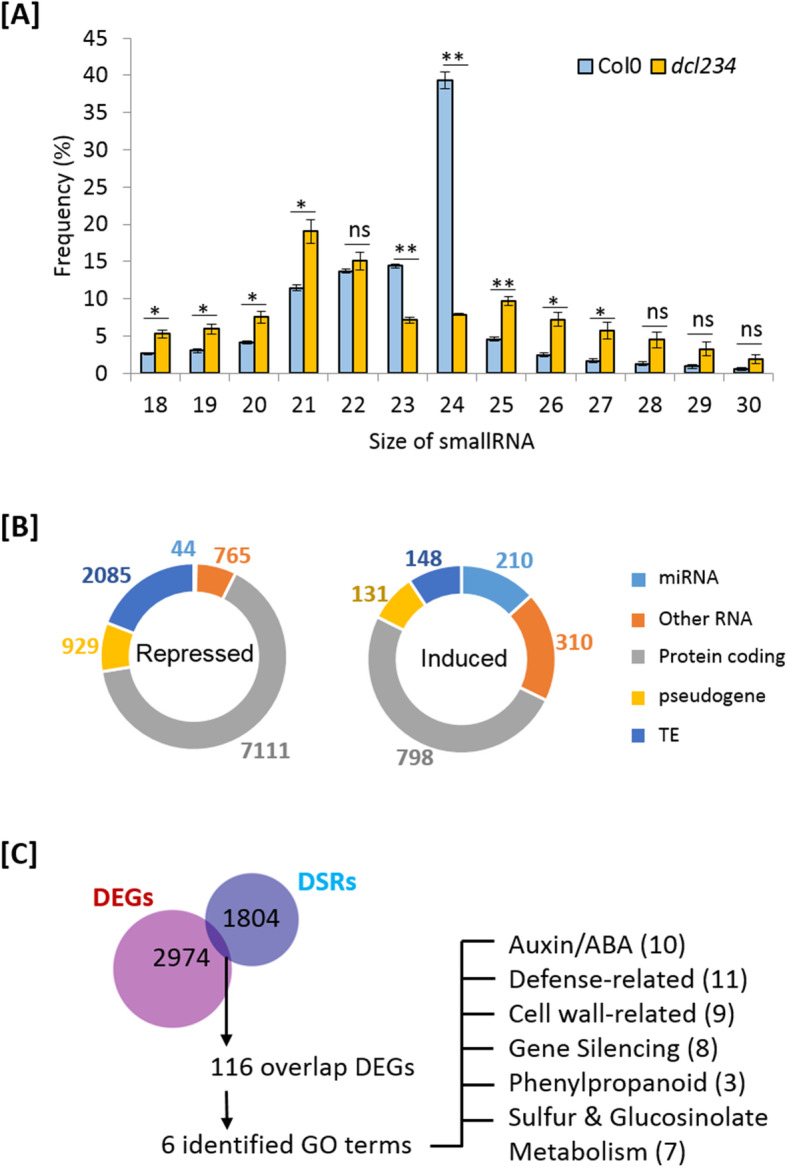
The *dcl234* mutant shows not only reduction but also ectopic accumulation of various sRNAs and indications of altered plant-microbe interactions. **a** Comparison of genome-wide sRNA abundance in *dcl234* and Col-0 regarding different sRNA sizes. Statistical significance of *p* ≤ 0.05 and *p* ≤ 0.01 (student *t* test) is indicated by * and **, respectively. Mean ± SE, *n* = 4. **b** The types of genetic loci where dcl234 shows differentially abundant sRNAs compared to Col-0. *TE* transposon. **c** GO analysis of DEGs that were associated with altered sRNA accumulation. *DEGs* differentially expressed genes, *DSRs* differential sRNA regions. See also Figure S7

To investigate the potential linkage between plant mRNA transcriptome and the sRNA homeostasis, we also profiled the sRNA transcriptome. Genome-wide sRNA sequencing detected a total of 107,307 sRNA in *dcl234* and Col-0, among which 63,971 (59.6%) and 15,901 (14.8%) showed reduced and increased abundance, respectively, in *dcl234* compared to Col-0 (Figure S7A). Sorting the sRNA population by sizes revealed that the reduction in sRNA levels was mainly contributed by 24 nt siRNAs and, to a less degree, by 23 nt siRNAs; in contrast, sRNA of 18-22 nt and 25–28 nt displayed increased levels in *dcl234* compared Col-0 (Fig. 3a; Table S5), possibly reflecting an accumulation of unprocessed substrates for DCLs 2, 3, and 4, as well as ectopic cleavage of the substrates by DCL1 that generates 21 nt miRNAs and by the RNase III-Like (RTL) proteins that may also cleave double-stranded RNAs [78]. Sorting the sRNA population by the types of genomic loci showed that the majority of differentially expressed sRNA originated from protein-coding gene regions, as well as highlighted a group of differentially expressed miRNAs, which are mostly upregulated in *dcl234* compared to Col-0 (Fig. 3b; Figure S7B). Altogether, these patterns demonstrate the broad impacts of the *dcl234* mutations on Arabidopsis sRNA homeostasis.

By connecting the analyses of both mRNA and sRNA, a group of 116 DEGs were identified as associated with alterations in sRNA abundance within the same loci (Fig. 3c; Table S6). GO analysis subsequently revealed gene enrichment in 6 biological processes, including defense, phenylpropanoid metabolism, sulfur and glucosinolate metabolism, auxin-/ABA-related, gene silencing, and cell wall-related (Fig. 3c). Because the phytohormones auxin and ABA both regulate root development, meanwhile the GO term cell wall was also highlighted in the *dcl234* downregulated DEGs, it became intriguing whether the *dcl234* mutations resulted in alterations in root architecture or cell wall composition, in addition to the defense-related processes.

### The *dcl234* mutations decrease cellulose and callose deposition in root xylem

**Fig. 4:**
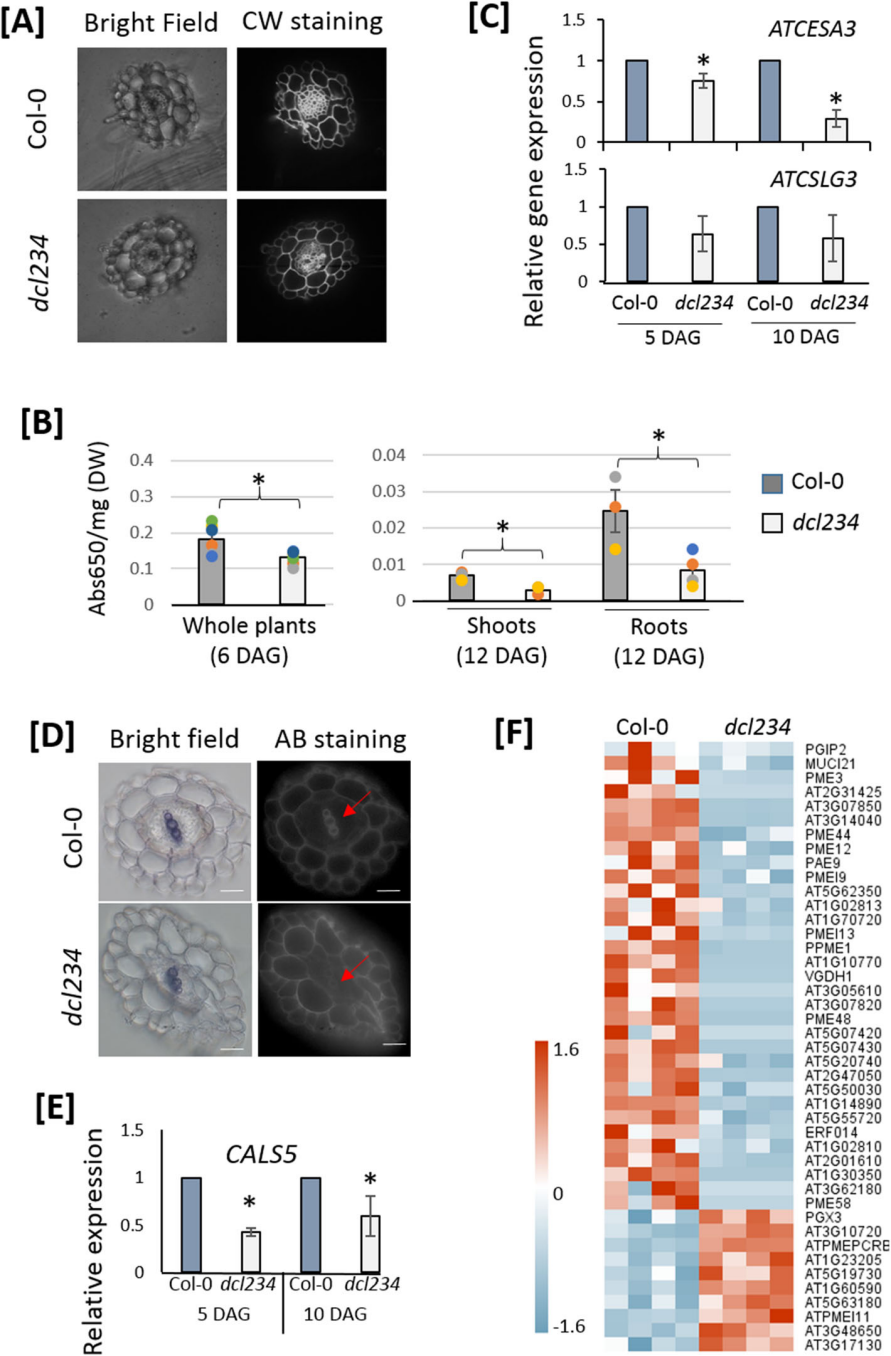
The *dcl234* mutant shows decreased deposition of cellulose and callose in root xylem. **a** Visualization of cellulose deposition by Calcofluor White (CW) staining. Transverse sections of primary roots from 12-day-old plants were compared. Representative images of 10 replicates are shown. **b** Quantification of cellulose contents in 6-day-old (*n* = 80 seedlings per biological replicate) whole seedlings and 12-day-old (*n* = 40 seedlings per biological replicate) shoots and roots. Mean ± SE, *n* = 3 biological replicates. * indicates *p* ≤ 0.05, student *t* test. **c** Quantitative RT-PCR measurements of Arabidopsis *CSLG3* and *CESA3* gene expression levels. Mean ± SE, *n* = 3 technical replicates. Two biological replicates were analyzed with similar results. * indicates *p* ≤ 0.05, student *t* test. **d** Visualization of callose deposition by Aniline blue (AB) staining. Red arrows point to the xylems where the callose levels are different in *dcl234* and Col-0. Transverse sections of primary roots from 12-day-old plants were compared. Representative images of 3 replicates are shown. **e** Quantitative RT-PCR measurements of Arabidopsis *CALS5* gene expression levels. Mean ± SE, *n* = 3 technical replicates. Two biological replicates were analyzed with similar results. * indicates *p* ≤ 0.05, student *t* test. **f** A heatmap of DEGs related to pectin homeostasis, as identified in RNAseq analysis. See also Figure S8

Following the transcriptional hints of altered cell wall composition, we next compared the roots of *dcl234* and Col-0 but found no morphological difference. However, microscopic visualization with Calcofluor White staining indicated that cellulose deposition was decreased in *dcl234* root xylem compared to Col-0 (Fig. 4a). Subsequently, quantitative measurements showed that the cellulose level was decreased by 28.5% in 6 day-after-germination (DAG) *dcl234* seedlings compared to Col-0 (Fig. 4b). Organ-specific measurements also revealed decreased cellulose levels in both roots and shoots in 12 DAG *dcl234* plants (Fig. 4b). In addition to the mRNA sequencing that identified a group of cellulose-related DEGs (Figure S8A), quantitative RT-PCR demonstrated repressed gene expression of cellulose synthase 3 (CESA3), which is a major cellulose synthase in Arabidopsis [79], and cellulose synthase-like G3 (CSLG3) in *dcl234* compared to Col-0 (Fig. 4c). CESA3 and CSLG3 are predicted targets of the miRNAs miR838 and miR395, respectively [58, 80], which were both identified as up-regulated in *dcl234* by the sRNA genome-wide analysis (Figure S7B). Together, these results suggest that the ectopic regulation of sRNA homeostasis in *dcl234* leads to the reductions in cellulose synthase gene expression and consequently cellulose levels.

In addition to cellulose, callose deposition in root xylem was also decreased in 12 DAG *dcl234* compared to Col-0, as indicated by Aniline Blue staining (Fig. 4d). Meanwhile, the callose synthase gene CALS5 was repressed by the *dcl234* mutations as measured at 5 and 10 DAG (Fig. 4e; Figure S8B). In Arabidopsis, callose deposition is positively regulated by miR160 and negatively regulated by miR398b and miR773 [81]. The *dcl234* mutations do not alter miR160 accumulation but increases the levels of miR398b and miR773 (Figure S7B). Thus, it appears that the decreased callose deposition in *dcl234* root xylem results from the ectopic expression of miRNA. In addition to cellulose and callose, the *dcl234* mutations may also disrupt the homeostasis of pectin, which represents a complex family of plant cell wall polysaccharides [82], as indicated by a group of 42 DEGs including pectin methyltransferases (PMEs) and PME inhibitors (PMEI) (Fig. 4f; Figure S8B; C). More indications of altered cell wall modification were also shown by differential expression of other cell wall-related genes, such as extensins that are important for root-microbe interactions and the peroxidases PRX40 and PRX9 that are known to crosslink extensins to maintain cell wall integrity [83, 84] (Table S4). Since cell wall plays a crucial role in plant interactions with microbes [85, 86], these results collectively suggest that the alterations in *dcl234* root microbiota may also be attributed to the altered plant cell wall modifications.

### The *dcl234* mutations alter the composition of root exudates

Root exudates create a nutrient-rich environment for microbes in the rhizosphere. To gain more insights into how the *dcl234* mutations influence root microbiota, we also investigated root exudates. Gas chromatography coupled with mass spectrometry (GC-MS) detected 15 and 2 root exudates components whose abundance was increased and decreased (*p* ≤ 0.05, student *t* test), respectively, in *dcl234* compared to Col-0 (Table 2; Figure S9A). Increased root secretion of boric acid and arabinose was observed. Given that the pectic polysaccharide rhamnogalacturonan II (RG-II) exists in primary cell walls as a dimer that is covalently cross-linked by a borate diester [87], and that arabinose is a constituent of many different cell wall components including RG-II [87], the increases in boric acid and arabinose in *dcl234* root exudates are consistent with an alteration in pectin metabolism, as was indicated by the pectin-related DEGs (Fig. 4f).

**Table 2:** Root exudates components that showed differential levels in dcl234 compared with Col-0. See also Figure S9

Compounds	RT (min)	Average Peak Area (*n* = 3)		Fold Change	*p*-value
		wt	dcl234		
Methylamine, 2TMS derivative	3.84	2379550	3816191	1.60	0.0422
Boric acid, 3TMS derivative	5.401	212521	559034	2.63	0.0101
Tetrasiloxane, decamethyl-	7.131	162751	1160090	7.13	0.0012
Tris(trimethylsilyl)amine	9.12	2558823	6037552	2.36	0.023
Oxalic acid	9.939	52953	109362	2.07	0.0302
Pentasiloxane, dodecamethyl-	10.92	9902	36314	3.67	0.0028
Benzoic Acid, TMS derivative	13.889	312624	638898	2.04	0.038
Silanol, trimethyl-, phosphate (3:1)	14.925	2292271	4810323	2.10	0.022
Glycerol, 3TMS derivative	15.106	71213	218472	3.07	0.00887
β-D-xylofuranose	21.785	18745	8789	0.47	0.001025
Ethylmethylsilyl-dipropylmalonate	24.486	7375	4651	0.63	0.018
D-Arabinose	31.14	3546	6807	1.92	0.034
β-D-Allopyranose	34.33	12259	43082	3.51	0.0201
Fructose/Sorbose	37.894	4414776	8097846	1.83	0.0311
Glucose/Mannose	39.203	1417682	2783229	1.96	0.0332
Inositol	44.659	3833826	6925326	1.81	0.0289
Sucrose	59.729	577625	1118469	1.94	0.0409

In addition to arabinose, several sugars including sucrose, glucose/mannose, and fructose/sorbose also showed higher levels in the *dcl234* root exudates, possibly correlated to the transcriptional up-regulation of photosynthesis-related processes and carbohydrate-related processes such as disaccharide biosynthesis (Fig. 2a; Table S4). The *dcl234* root exudates also contained more allopyranose, which is a rare sugar that suppresses gibberellin (GA) signaling in rice [88]; consistently, the *dcl234* transcriptome analysis revealed a group of GA-related DEGs that were all down-regulated (Figure S9B). The transcriptome analysis identified a group of amine biosynthesis genes as upregulated DEGs in *dcl234* (Table S4); consistently, *dcl234* root exudates contained higher levels of silanamine and methylamine compared to Col-0. The increased production of methylamine in *dcl234* is consistent with the increased enrichment of *methylophilaceae*, which are methylotrophic bacteria that consume methylamine [89], within the *dcl234* root microbiota (Figure S4B; Table S3).

Consistent with the transcriptional upregulation of phenylpropanoid biosynthesis (Table S4; Figure S6B), the *dcl234* root exudates showed a higher level of benzoic acid, which is a phenylpropanoid compound that can serve as a precursor of SA biosynthesis [90]. Thus, benzoic acid may coordinate the simultaneous activation of defense responses and phenylpropanoid biosynthesis in *dcl234*. Interestingly, gene expression of BAH1, a negative regulator of SA production [90], was repressed in *dcl234*; meanwhile, miR827 that targets BAH1 was induced in *dcl234* (Figure S9C), thereby providing a possible linkage between the transcriptional regulation of defense responses and the ectopic expression of miRNA caused by the *dcl234* mutations. The content of glycerol in root exudates was increased by 3.1-fold in *dcl234* compared to Col-0. Exogenous application of glycerol induces defense responses in Arabidopsis and impairs plant responses to beneficial bacteria [91]. In addition, Arabidopsis infected by the pathogen *Pst* DC3000 accumulated higher levels of glycerol [92]. Thus, the increased accumulation of glycerol in *dcl234* root exudates is in accordance with the activated plant defense responses.

## Discussion

Arabidopsis DCL2 and DCL4 are involved in antiviral defense through processing viral double-stranded RNAs into 21 nt or 22 nt sRNAs [29, 93]. In addition, DCL4 and DCL3 both negatively regulate the expression of some NLRs in the absence of pathogen infection [30]. When grown in the soil that contained various types of microbes, the *dcl234* mutant showed transcriptional activation of defense-related genes compared to the wild-type plants, further supporting the importance of these DCL proteins in plant defense responses. This would be tested in future research that compares Col-0 and *dcl234* under soil-grown and axenically grown conditions. The functions of DCL2, DCL3, and DCL4 in mediating plant disease resistance are partially redundant, because the single mutants generally showed no or very weak difference compared to the wild-type plants when infected by pathogens, whereas altered disease symptoms were mainly observed in the DCL double or triple mutants [30]. Similarly, the *dcl234* mutant showed more obvious epinastic leaves compared to any *dcl* single or double mutants, suggesting that the three DCL proteins are parts of a continuum in regulating plant physiology [28, 29].

Plants activate defense responses when cell wall integrity is altered by genetic or physical disruption of cell wall biosynthesis and/or remodeling [85, 94]. Thus the activation of defense-related genes in *dcl234* may also be attributed to the alterations in cell wall, in addition to being connected with certain metabolites such as the root exudate component benzoic acid. The *dcl234* triple mutation not only drastically reduces the levels of 24 nt and 23 nt siRNAs but also results in ectopic increases in the levels of other sRNAs. In Arabidopsis, 24 nt siRNAs account for most of the genome-wide siRNAs and are mainly DCL3-dependent. This is consistent with the observation that, on a whole-genome scale, there are more sRNAs that showed decreased abundance than sRNAs that showed increased abundance. *Arabidopsis thaliana* encodes nine RNase III, including four DCLs and five RTLs, which cleave double-stranded RNAs [78]. On the one hand, the ectopic accumulation of sRNAs, such as 21 nt sRNAs that are mainly DCL1-dependent, indicates that the substrates of DCL2, DCL3, and DCL4 may be alternatively cleaved by DCL1 and/or RTLs in the *dcl234* mutant. On the other hand, the ectopic accumulation of sRNAs can also be attributed to the unprocessed substrates of DCL2, DCL3, and DCL4. In fact, the *dcl234* triple mutation has been shown to cause a significant accumulation of Pol IV-produced noncoding RNAs, which are mostly 26–50 nt RNAs that are transcribed by RDR2 into double-stranded RNA and subsequently processed mainly by DCL3 into 24 nt siRNAs [95–99]. Certain double-stranded RNAs can trigger plant defense responses in a way that is independent on DCL proteins [100], thus it is likely that the ectopic accumulation of DCL3-, DCL2-, and DCL4-substrates is another reason for the activated defense responses in the *dcl234* mutant.

In the canonical RdDM pathway, the plant-specific RNA polymerases Pol IV and Pol V are two core components; the former controls siRNA production while the latter controls scaffold RNA production. Thus in theory, *nrpd1* (Pol IV mutant) and *nrpe1* (Pol V mutant) are already sufficient for a conclusion about function of the canonical RdDM in any biological processes including root microbiota assemblage. In this study, we used *rrp6l1*, *dcl234*, and *ddc* in addition to *nrpd1* and *nrpe1*. Rrp6L1 mediates chromatin retention of non-coding RNAs, while DCL2/3/4 proteins cleave precursor RNAs to generate siRNAs. Because RdDM is not the only function of non-coding RNAs (including siRNAs), we used *rrp6l1* and *dcl234* to see whether these two mutants would have different impacts (in addition to the common impacts due to RdDM) on root microbiota compared to *nrpd1* and *nrpe1*. The mutant *ddc* is defective in the DNA methylases DRM1, DRM2, and CMT3. If there were any alterations in root microbiota in *nrpd1* and *nrpe1* compared to wild-type plants, *ddc* would allow us to determine whether the observed effects were caused by changes in DNA methylation or by the up-stream non-coding RNAs. Therefore, our mutant combination was sufficient and reasonable for the research purpose. Small RNAs control many important biological processes through either epigenetic regulation of the genome or post-transcriptional regulation of the transcriptome. Although DCL-dependent siRNA function in mediating canonical RdDM, our result indicate that this epigenetic function does not contribute to the impacts of the *dcl234* mutations on root microbiota, because dysfunction of the other key regulators of RdDM, such as Pol IV and Pol V, did not alter root microbiota. However, it should be noted that the *dcl234* mutations may also cause ectopic accumulation of new siRNA and consequently result in ectopic RdDM activities that may influence root interactions with soil microbes.

## Conclusions

**Fig. 5:**
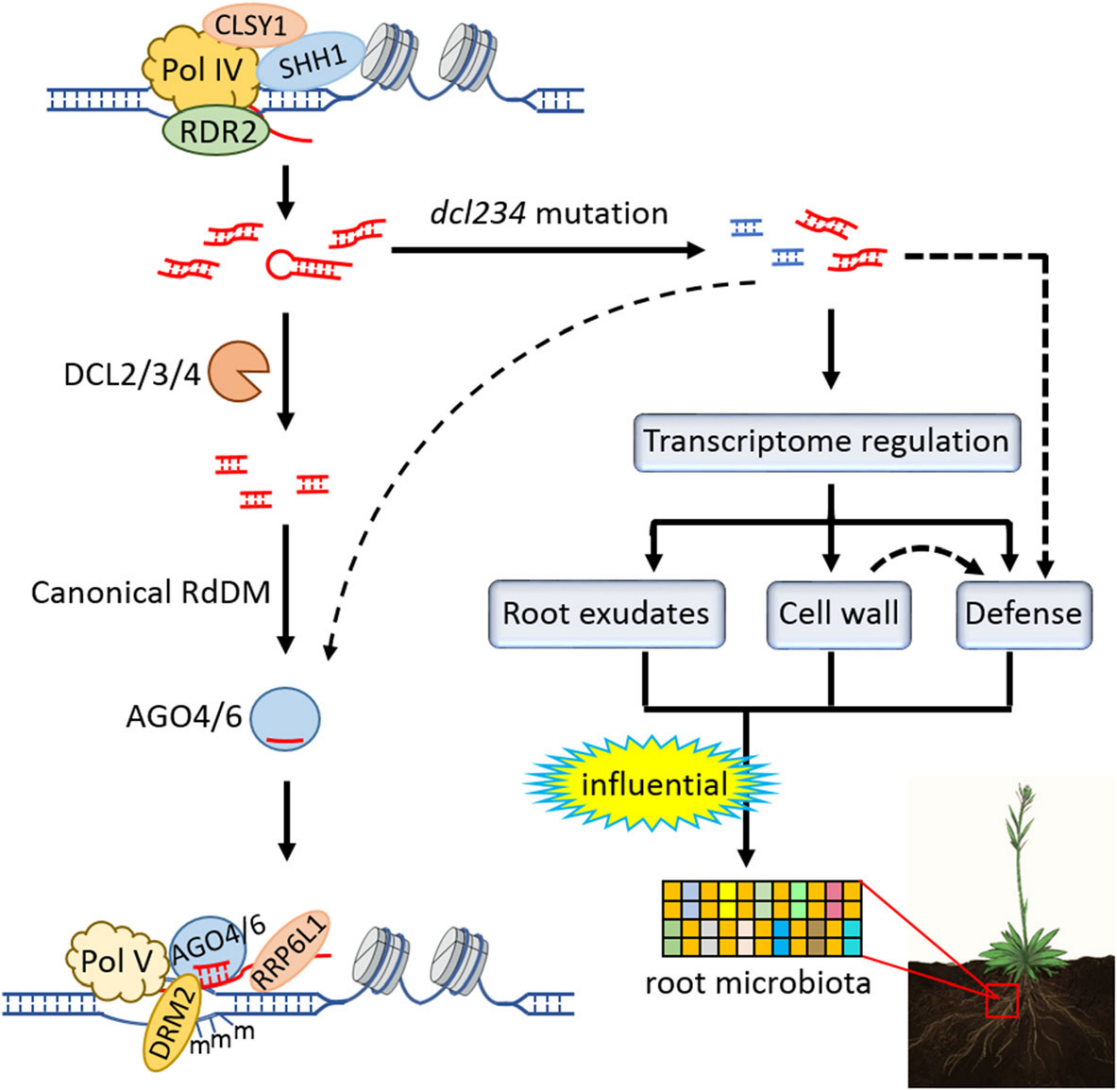
A schematic model depicting the functional mechanism underlying the impacts on root microbiota by the *dcl234* triple mutation. In the canonical RdDM pathway, Arabidopsis DCL2, DCL3, and DCL4 function in continuum to regulate the biogenesis of 21–24 nt siRNAs, whose precursors originate from Pol IV-dependent transcription. In wild-type Arabidopsis, the canonical RdDM pathway shows no connection with the assembly of root microbiota. In the *dcl234* triple mutant, the disrupted sRNA homeostasis leads to multilayered alterations in several biological processes that are important for plant-microbe interactions, including defense-related gene expression, cell wall modifications, and root exudation of metabolites, which are concomitant with an altered root microbiota assembly. Dashed arrows indicate potential effects (see the main text for details). Besides the *dcl234* triple mutant, the RdDM mutants (with the corresponding gene names in parentheses) investigated in this study include *nrpd1*-*3* (Pol IV), *nrpe1*-*11* (Pol V), *rrp6l1*-*2* (RRP6L1), and *ddc* (DRM1, DRM2, CMT3). In addition to the examined factors, several other RdDM components are also shown [24], including CLSY1 (CLASSY 1), SHH1 (SAWADEE HOMEODOMAIN HOMOLOGUE 1), RDR2 (RNA-DEPENDENT RNA POLYMERASE 2), AGO4 (ARGONAUTE 4), and AGO6. For simplicity, not all known RdDM components are shown

Plants naturally associate with a diverse community of soil microbes. Important questions are emerging regarding potential linkages between the assembly of root microbiota and key cellular processes in the plant. DCL proteins regulate the biogenesis of small RNAs, which are key mediators of many biological processes including epigenetic modifications such as RNA-directed DNA methylation (RdDM). In this study, we demonstrate an important role of the DCL proteins in influencing root microbiota, which is characterized by drastic reductions in the relative abundance of *Aeromonadaceae* and *Pseudomonadaceae*. Our investigations further revealed that the DCL proteins confers integrated regulation of plant defense, cell wall compositions, and root exudates, all of which are important factors that influence plant-microbe interactions, thereby providing mechanistic insights into the important function of the DCL proteins in regulating root microbiota (Fig. 5).

Unlike *dcl234*, the other examined RdDM mutants showed similar root microbiota compared to the wild-type plants. Therefore, our results also demonstrate that the DCL proteins regulate root microbiota independently of their functions in mediating RdDM, and that the canonical RdDM is dispensable for Arabidopsis root microbiota (Fig. 5). Altogether, these findings not only establish a connection between root microbiota and plant epigenetic factors but also highlight the complexity of plant regulation of root microbiota.

## Supplementary Information

**Additional file 1: Figure S1.** The largest separation between bacterial communities is spatial proximity to the root as revealed by PCoA plotted from wUniFrac metrics (Related to Fig. 1). [A] PCo 1 vs PCo 2. [B] PCo 2 vs PCo 3. [C] PCo 1 vs PCo 3. **Figure S2.** Microbe richness in different compartments reflects the selectivity of plants on root-associated microbes (Related to Fig. 1). [A] Numbers of observed OTUs in the different compartments. [B] Numbers of estimated OTUs based on the Chao1 estimator. [C] Shannon index of the microbe richness. Samples were rarefied to 23000 reads prior to the analysis. Soil1 is the initial bulk soil and Soil2 is the final bulk soil. Letters denote statistical significance (p ≤ 0.05, Wilcox test) compared to Soil 1. **Figure S3.** Taxonomic structure of Abundant Community members (ACM) is affected by compartments and plant genotype (Related to Fig. 1). [A] Relative abundance (RA) of the bacteria within the initial bulk soil (Soil 1) and the final bulk soil (Soil 2) as classified at the phylum level. [B] Relative abundance of the bacteria phyla that were identified within the rhizosphere samples. [C] Relative abundance of the bacteria phyla that were identified within the root samples. **Figure S4.** The *dcl234* triple mutation alters Arabidopsis root microbiota (Related to Fig. 1). [A] Relative abundance of the top 5 abundant phyla in roots of the wild type Arabidopsis (Col-0) and the RdDM pathway mutants. Mean ± SE, n ≥ 3. Asterisks indicate significant difference (FDR ≤ 0.05) between the mutant and the wild type. Taxa with RA > 5% in at least one sample were included in the statistical analysis. [B] A heatmap showing the levels of OTUs with significantly different enrichment (FDR ≤ 0.05) in *dcl234* compared to Col-0. Phyla are annotated on the left side of the heatmap; on the right side, OTUs are annotated to different levels, F, family, G, genus, O, order; NR, New Reference. **Figure S5.** Read counts of the metagenomic sequencing. Stacked bars are shown to indicate the read counts of both plant DNA and microbial DNA sequences in each sample. Mean ±SE, n = 4. * indicates p ≤ 0.05; NS, non-significant, student t-test. **Figure S6.** The *dcl234* triple mutation causes alterations in Arabidopsis defense-related processes (Related to Fig. 2). [A] A heatmap of DEGs involved in phenylpropanoid production. [B] Root anthocyanin visualization and measurements in plants at 18 days after germination. Mean ± SE, n = 6 biological replicates. Each biological replicate consisted of roots from 3 plants. **, p ≤ 0.01, student t-test. [C] A heatmap of DEGs involved in sulfur metabolism. DEGs were identified in the mRNAseq transcriptome analysis. The two heatmaps use the same Z-Score color scale that is shown in panel C. **Figure S7.** The *dcl234* mutant shows both decreased and increased accumulation of different sRNAs (Related to Fig. 3). [A] The MA plot of the sRNA population identified in Col-0 and dcl234. Y-axis displays fold changes (*dcl234* vs Col-0) of sRNA abundance; X-axis displays average signal intensities of the sRNA in all samples. The red, purple, and black colors indicate sRNAs that were increased, decreased, or not changed in *dcl234* compared to Col-0 based on FDR p ≤ 0.05. [B] A heatmap of miRNAs identified as increased or decreased in *dcl234* compared to Col-0. [C] Examples of genetic loci where sRNA abundance was affected by the *dcl234* triple mutation. Snapshot images were obtained from whole-genome sRNA sequencing results. Vertical gray bars indicate the sRNA sequencing coverage normalized to the same scale in Col-0 and *dcl234*. **Figure S8.** The *dcl234* mutant shows altered expression of cell wall-associated genes (Related to Fig. 4). [A] A heatmap of DEGs related to cellulose synthesis. [B] Expression levels of DEGs related to callose synthesis and pectin metabolism. Snapshot images were obtained from whole-genome sRNA sequencing results. Vertical gray bars indicate the mRNA sequencing coverage normalized to the same scale in Col-0 and *dcl234*. [C] Quantitative RT-PCR measurements of two pectin-related DEGs. Mean ± SE, n=3 technical replicates. Two biological replicates were analyzed with similar results. * indicates p ≤ 0.05, student t-test. **Figure S9.** The *dcl234* mutant shows altered transcription regulation of metabolism that potentially connects to alterations in root exudates (Related to Table 2). [A] Gas Chromatography-Mass Spectrometry (GC-MS) analysis of root exudates from Col-0 and *dcl234*. Representative profiles were shown. n =3 biological replicates. [B] A heatmap of gibberellin-related DEGs. [C] Expression levels of miR827 and its target gene BAH1. Snapshot images were obtained from whole-genome sRNA and mRNA sequencing results. Vertical gray bars indicate the sequencing coverage normalized to the same scale in Col-0 and *dcl234*.

**Additional file 2: Table S1.** Sample information and the indexing and barcode details of 16S rRNA gene amplification.

**Additional file 3: Table S2.** Primers used in this study.

**Additional file 4: Table S3.** Taxonomic structure of abundant community members (ACMs) based on relative abundance (RA) at family and phylum levels in RdDM pathway mutants as compared to Col-0.

**Additional file 5: Table S4.** RNAseq profiling of dcl234 in comparision to Col-0 and GO annotation of UP and Down regulated DEGs.

**Additional file 6: Table S5.** Distribution of smallRNA in dcl234 and Col-0.

**Additional file 7: Table S6.** Common loci associated with smallRNA generation and differential expression of transcripts.

## Data Availability

The datasets generated and/or analyzed during the current study are available at following repositories. The mRNASeq and sRNA sequencing data from this publication have been deposited to the NCBI Gene Expression Omnibus (http://www.ncbi.nlm.nih.gov/geo/) and are accessible through GEO Series accession number GSE148083. The 16S rRNA gene sequencing and metagenome sequencing data have been deposited to the NCBI SRA database (https://www.ncbi.nlm.nih.gov/sra) and are accessible through the accession number PRJNA622888. The tokens for GSE148083 is qpcbeysyzlyrrgb.
