# Supplementary material for: Dicer-like proteins influence Arabidopsis root microbiota independent of RNA-directed DNA methylation
Source: Microbiome. 2021 Feb 26;9:57. doi: 10.1186/s40168-020-00966-y (PMC7913254; doi:10.1186/s40168-020-00966-y)
Supplement: Supplementary file 2 — Additional file 1: Figure S1. The largest separation between bacterial communities is spatial proximity to the root as revealed by PCoA plotted from wUniFrac metrics (Related to Fig. 1). [A] PCo 1 vs PCo 2. [B] PCo 2 vs PCo 3. [C] PCo 1 vs PCo 3. Figure S2. Microbe richness in different compartments reflects the selectivity of plants on root-associated microbes (Related to Fig. 1). [A] Numbers of observed OTUs in the different compartments. [B] Numbers of estimated OTUs based on the Chao1 estimator. [C] Shannon index of the microbe richness. Samples were rarefied to 23000 reads prior to the analysis. Soil1 is the initial bulk soil and Soil2 is the final bulk soil. Letters denote statistical significance (p ≤ 0.05, Wilcox test) compared to Soil 1. Figure S3. Taxonomic structure of Abundant Community members (ACM) is affected by compartments and plant genotype (Related to Fig. 1). [A] Relative abundance (RA) of the bacteria within the initial bulk soil (Soil 1) and the final bulk soil (Soil 2) as classified at the phylum level. [B] Relative abundance of the bacteria phyla that were identified within the rhizosphere samples. [C] Relative abundance of the bacteria phyla that were identified within the root samples. Figure S4. The dcl234 triple mutation alters Arabidopsis root microbiota (Related to Fig. 1). [A] Relative abundance of the top 5 abundant phyla in roots of the wild type Arabidopsis (Col-0) and the RdDM pathway mutants. Mean ± SE, n ≥ 3. Asterisks indicate significant difference (FDR ≤ 0.05) between the mutant and the wild type. Taxa with RA > 5% in at least one sample were included in the statistical analysis. [B] A heatmap showing the levels of OTUs with significantly different enrichment (FDR ≤ 0.05) in dcl234 compared to Col-0. Phyla are annotated on the left side of the heatmap; on the right side, OTUs are annotated to different levels, F, family, G, genus, O, order; NR, New Reference. Figure S5. Read counts of the metagenomic sequencing. Stacked bars a [file 40168_2020_966_MOESM2_ESM.docx]

**
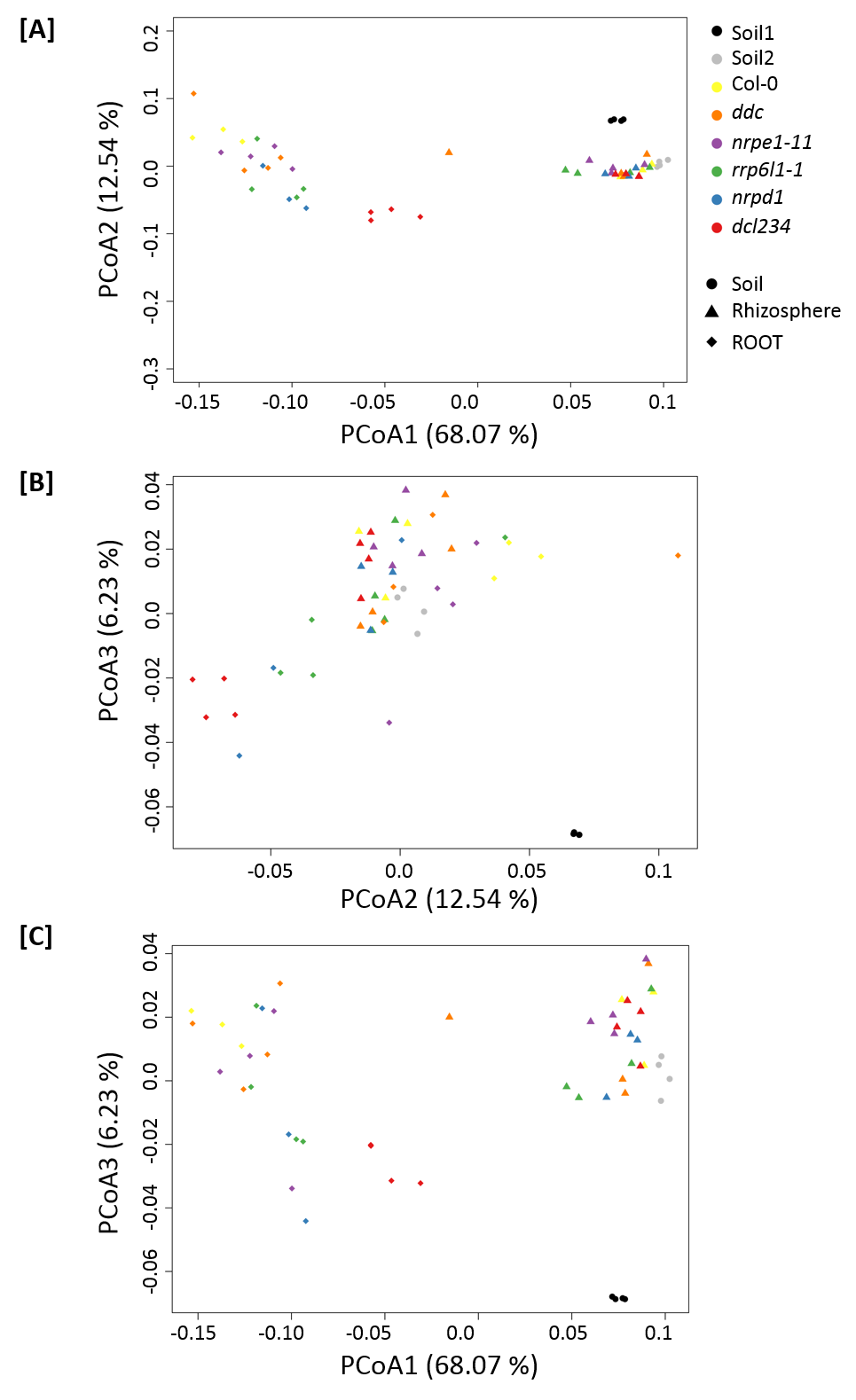
**

**Figure S1. The largest separation between bacterial communities is spatial proximity to the root as revealed by PCoA plotted from wUniFrac metrics (Related to Figure 1)**


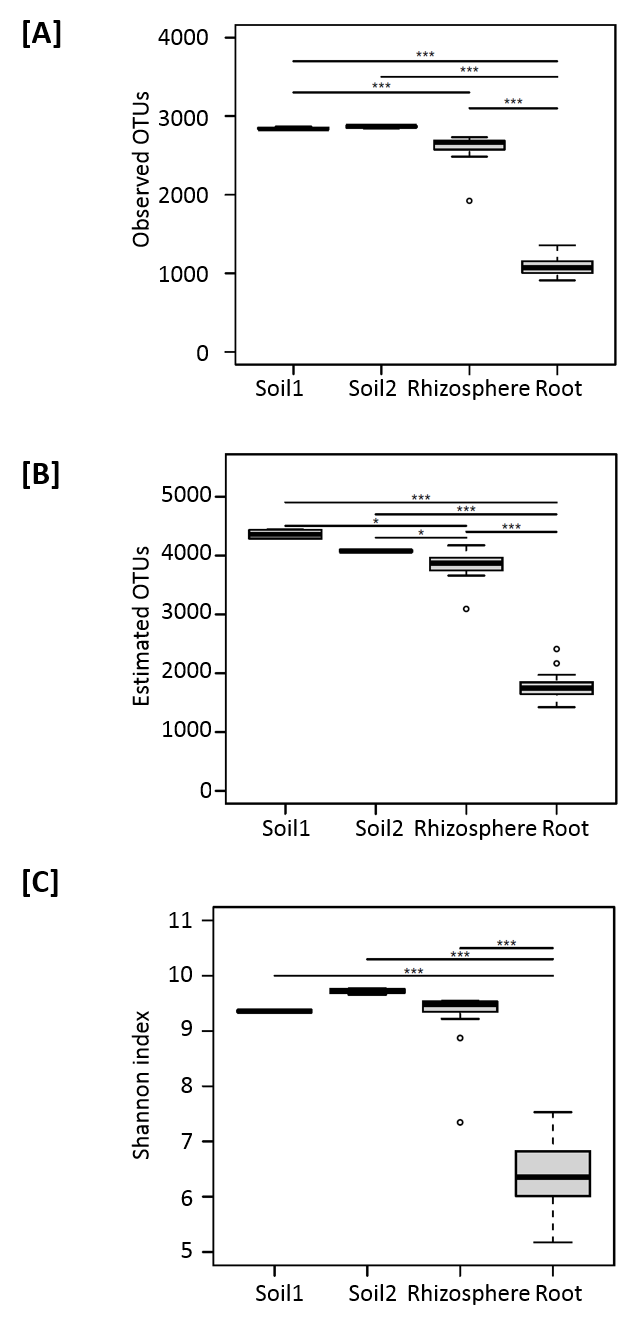


**Figure S2. Microbe richness in different compartments reflects the selectivity of plants on root-associated microbes (Related to Figure 1)**


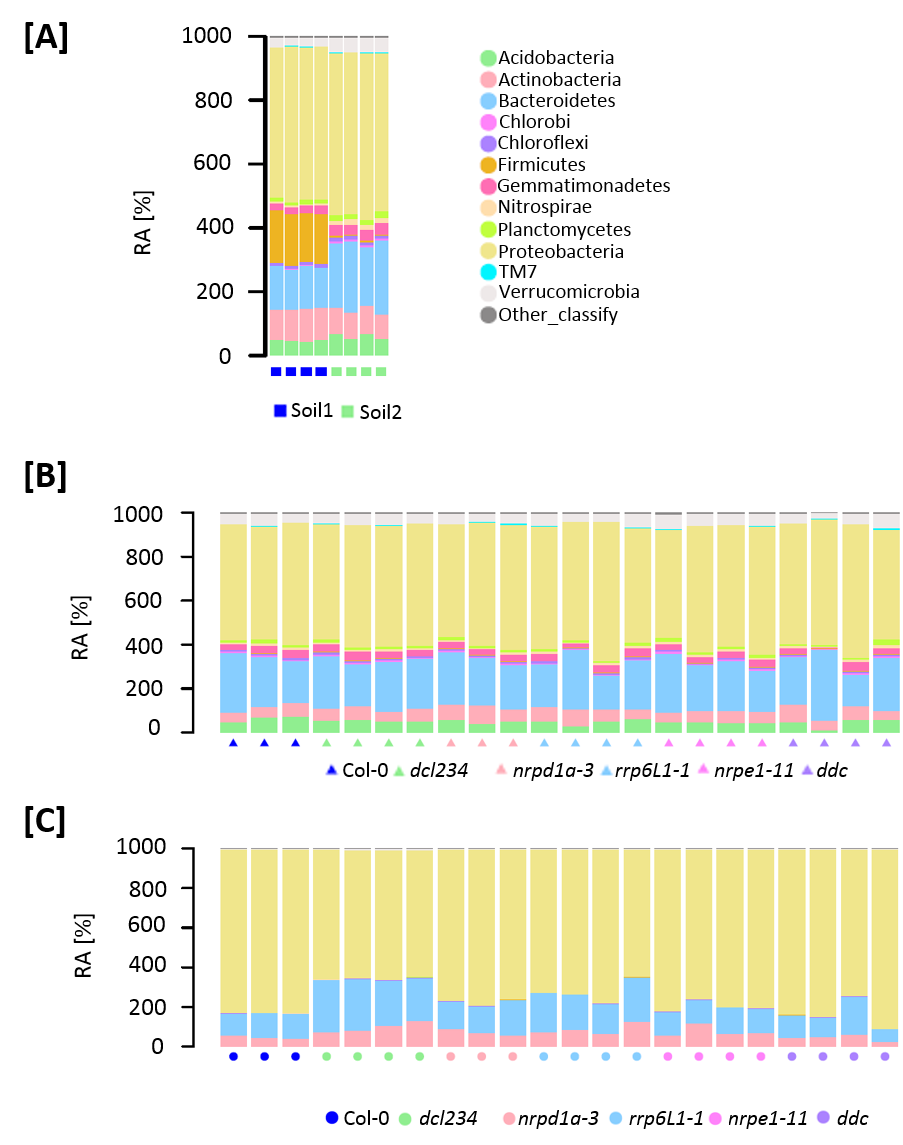


**Figure S3. Taxonomic structure of Abundant Community members (ACM) is affected by compartments and plant genotype (Related to Figure 1)**


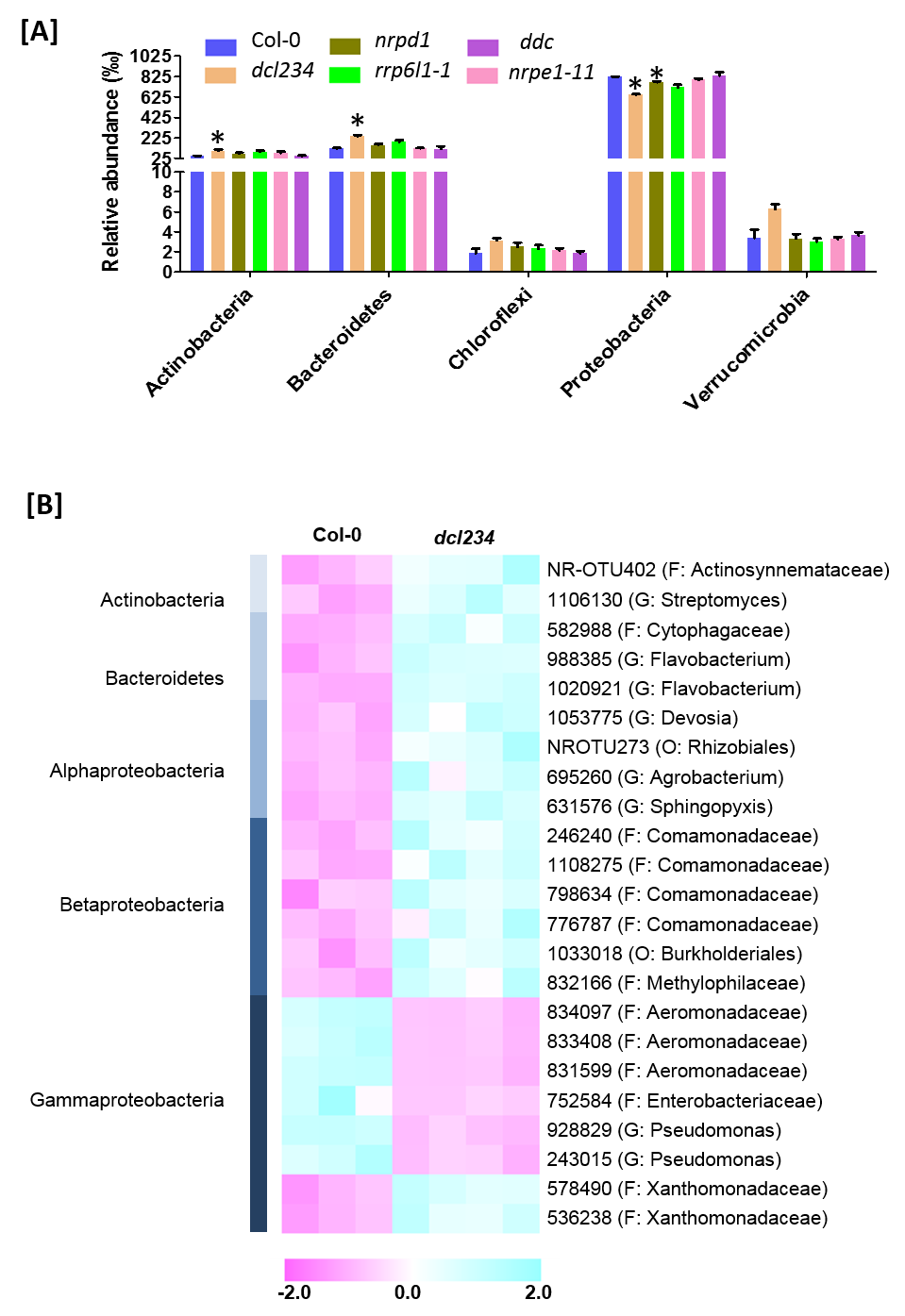


**Figure S4. The *dcl234* triple mutation alters Arabidopsis root microbiota (Related to Figure 1)**


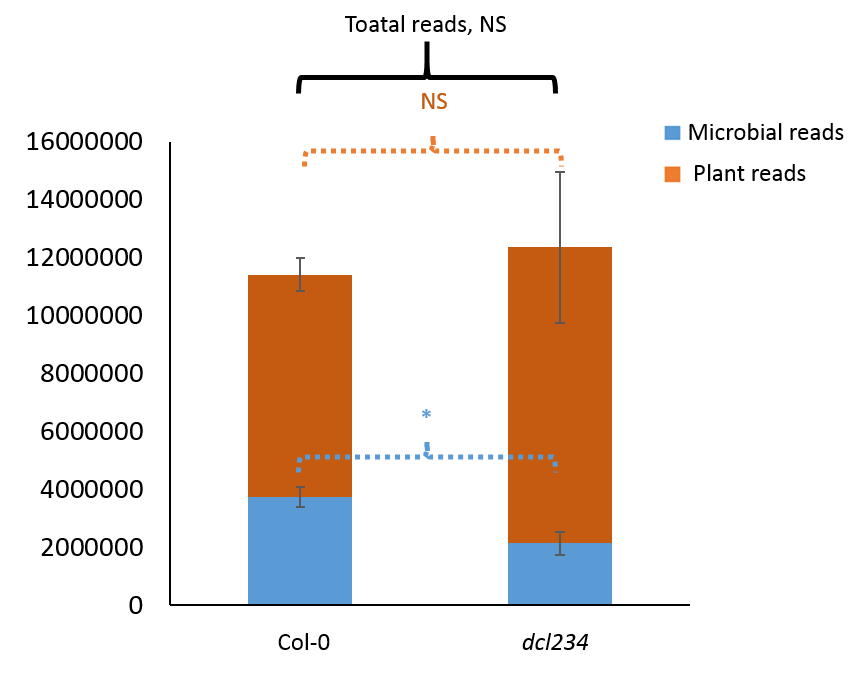


**Figure S5. Read counts of the metagenomic sequencing.**


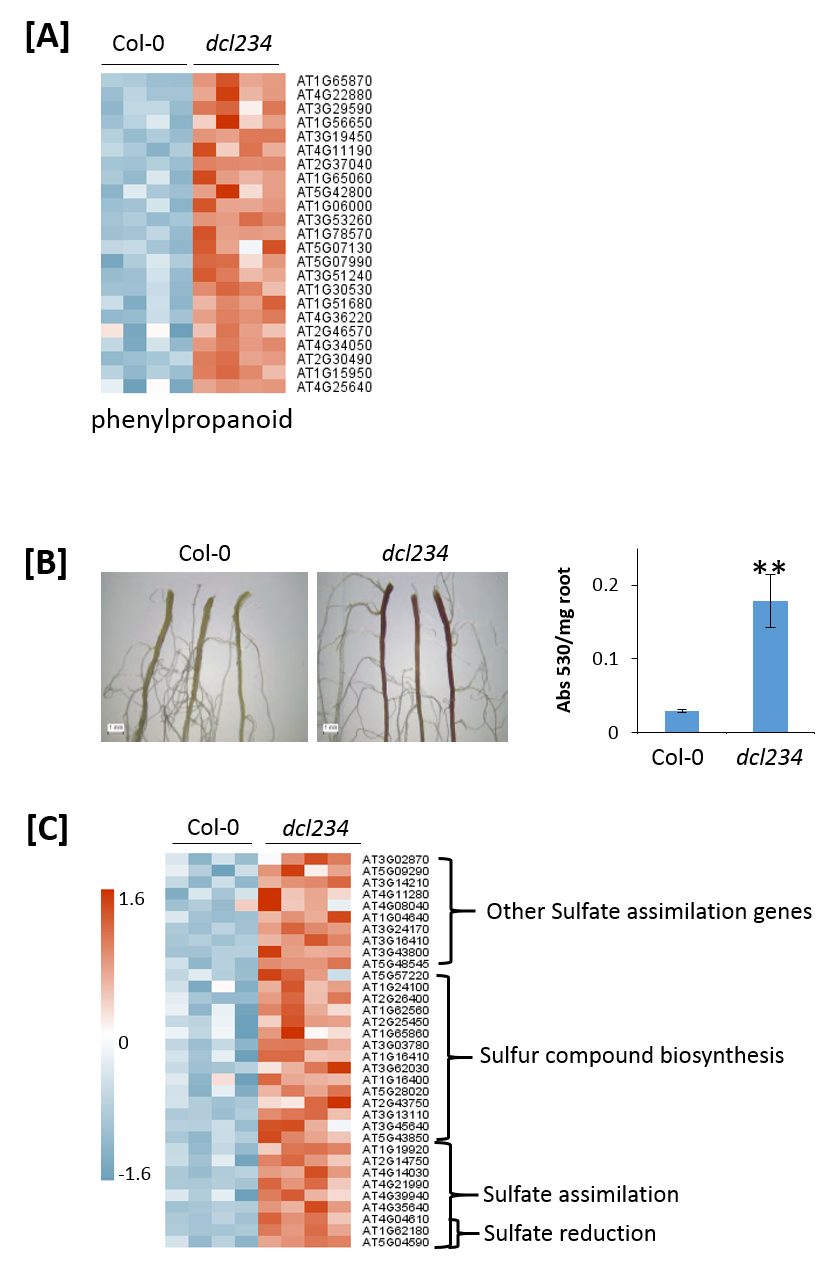


**Figure S6. The *dcl234* triple mutation causes alterations in Arabidopsis defense-related processes (Related to Figure 2)**


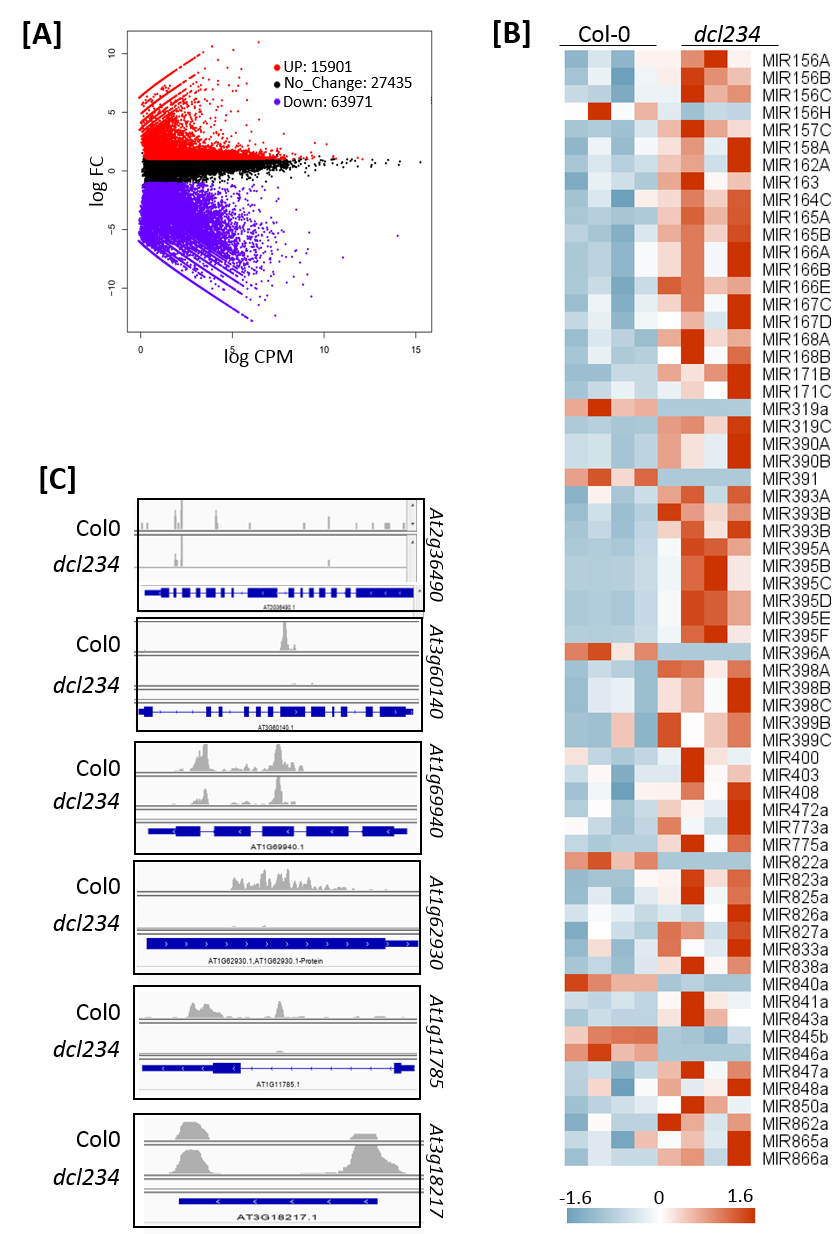


**Figure S7. The *dcl234* mutant shows both decreased and increased accumulation of different sRNAs (Related to Figure 3)**


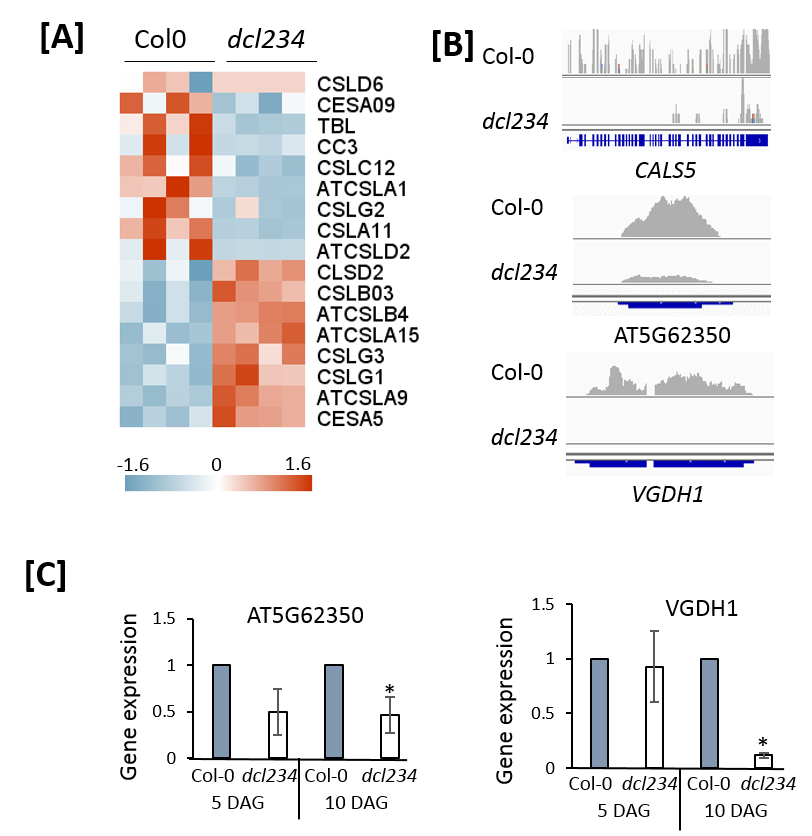


**Figure S8. The *dcl234* mutant shows altered expression of cell wall-associated genes (Related to Figure 4)**


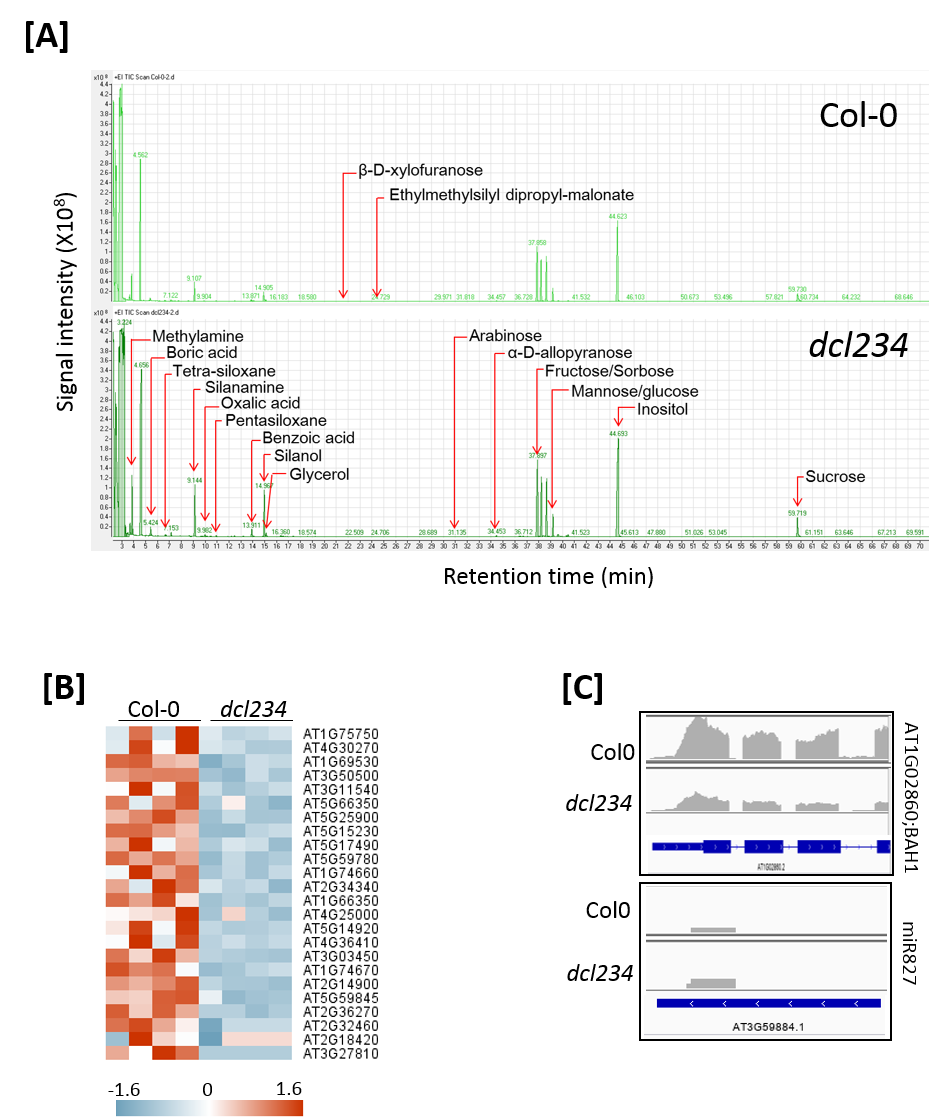


**Figure S9. The *dcl234* mutant shows altered transcription regulation of metabolism that potentially connects to alterations in root exudates (Related to Table 2)**
